# Comparative safety of tramadol and other opioids following total hip and knee arthroplasty

**DOI:** 10.1186/s12877-024-04933-2

**Published:** 2024-04-05

**Authors:** Elliott Bosco, Melissa R. Riester, Francesca L. Beaudoin, Andrew J. Schoenfeld, Stefan Gravenstein, Vincent Mor, Andrew R. Zullo

**Affiliations:** 1 Department of Health Services, Policy, and Practice, Brown University School of Public Health, Providence, RI USA; 2 Center for Gerontology and Healthcare Research, Brown University School of Public Health, Providence, RI USA; 3 Department of Epidemiology, Brown University School of Public Health, Providence, RI 02912 USA; 4 Department of Emergency Medicine, Brown University Warren Alpert Medical School, Providence, RI USA; 5 Department of Orthopaedic Surgery, Brigham and Women’s Hospital, Harvard Medical School, Boston, MA USA; 6 Center of Innovation in Long-Term Services and Supports, Providence Veterans Affairs Medical Center, Providence, RI USA; 7 Department of Medicine, Brown University Warren Alpert Medical School, Providence, RI USA

**Keywords:** Arthroplasty, Geriatrics, Tramadol, Oxycodone, Hydrocodone

## Abstract

**Background:**

Tramadol is increasingly used to treat acute postoperative pain among older adults following total hip and knee arthroplasty (THA/TKA). However, tramadol has a complex pharmacology and may be no safer than full opioid agonists. We compared the safety of tramadol, oxycodone, and hydrocodone among opioid-naïve older adults following elective THA/TKA.

**Methods:**

This retrospective cohort included Medicare Fee-for-Service beneficiaries ≥ 65 years with elective THA/TKA between January 1, 2010 and September 30, 2015, 12 months of continuous Parts A and B enrollment, 6 months of continuous Part D enrollment, and no opioid use in the 6 months prior to THA/TKA. Participants initiated single-opioid therapy with tramadol, oxycodone, or hydrocodone within 7 days of discharge from THA/TKA hospitalization, regardless of concurrently administered nonopioid analgesics. Outcomes of interest included all-cause hospitalizations or emergency department visits (serious adverse events (SAEs)) and a composite of 10 surgical- and opioid-related SAEs within 90-days of THA/TKA. The intention-to-treat (ITT) and per-protocol (PP) hazard ratios (HRs) for tramadol versus other opioids were estimated using inverse-probability-of-treatment-weighted pooled logistic regression models.

**Results:**

The study population included 2,697 tramadol, 11,407 oxycodone, and 14,665 hydrocodone initiators. Compared to oxycodone, tramadol increased the rate of all-cause SAEs in ITT analyses only (ITT HR 1.19, 95%CLs, 1.02, 1.41; PP HR 1.05, 95%CLs, 0.86, 1.29). Rates of composite SAEs were not significant across comparisons. Compared to hydrocodone, tramadol increased the rate of all-cause SAEs in the ITT and PP analyses (ITT HR 1.40, 95%CLs, 1.10, 1.76; PP HR 1.34, 95%CLs, 1.03, 1.75), but rates of composite SAEs were not significant across comparisons.

**Conclusions:**

Postoperative tramadol was associated with increased rates of all-cause SAEs, but not composite SAEs, compared to oxycodone and hydrocodone. Tramadol does not appear to have a superior safety profile and should not be preferentially prescribed to opioid-naïve older adults following THA/TKA.

**Supplementary Information:**

The online version contains supplementary material available at 10.1186/s12877-024-04933-2.

## Introduction

Pain management after total hip and knee arthroplasty (THA/TKA) is a critical part of postoperative care [1]. Moderate to severe pain is typical and pain control is important to participate in rehabilitation activities and restore function. Opioid analgesics are commonly prescribed for acute postoperative pain. However, given the risk of dependence with long-term use, postoperative pain management has shifted toward opioid-sparing regimens [1]. Tramadol is a serotonin–norepinephrine reuptake inhibitor (SNRI) and prodrug that is metabolized into a weak partial opioid agonist [2]. As a result of its unique pharmacology, tramadol is perceived by many clinicians to be safer than full agonist opioids like oxycodone or hydrocodone and to even be “opioid-sparing” by reducing exposure to full opioid agonist drugs. The Drug Enforcement Administration classifies tramadol as a Schedule IV controlled substance, indicating that it is perceived to have a lower risk of addiction and misuse than Schedule II opioids like oxycodone and hydrocodone. Tramadol has therefore become increasingly popular for postoperative pain management [3].

While use of tramadol has increased substantially over time [4–7], it is a potentially risky and unpredictable drug due to a number of factors. As a prodrug, it must be metabolized from an SNRI into an opioid metabolite by the CYP2D6 enzymes in the liver. The ability to metabolize drugs through CYP2D6 varies significantly between individuals. Slower metabolizers will experience the serotonin and norepinephrine (antidepressant) effects of tramadol with very little opioid analgesia, while fast metabolizers can experience more opioid analgesia than intended by prescribers. Age-related changes to drug metabolism create more variation in metabolism of tramadol among older adults, as does use of multiple chronic medications requiring metabolism through the CYP2D6 enzymes. Tramadol’s effects are therefore unpredictable and could potentially result in more adverse events than other opioids following surgery [8]. Further, it is unclear how the real-world safety of tramadol compares to other opioids when used following high-intensity interventions, such as THA/TKA, among older adults [9]. Given the heightened risk of peri-operative morbidity in this vulnerable population, it is critical to ensure that efforts to minimize full opioid agonist exposure through the use of tramadol do not come at the expense of increased adverse events and worse health outcomes.

We assessed the safety of tramadol compared to other opioids commonly prescribed to older adults for postoperative pain following THA/TKA using national data. We hypothesized that use of tramadol would result in higher risks of all-cause and serious adverse events when compared to oxycodone or hydrocodone.

## Methods

### Study design and data sources

This retrospective cohort study leveraged data from the Medicare Master Beneficiary Summary File; Medicare Provider Analysis and Review (MedPAR) inpatient claims; a 20% random sample of Medicare Part B (outpatient services) and Part D (prescription drugs) claims. The timing and location of health services utilization was determined using the validated Residential History File [10]. All sources included data from January 1, 2009 to December 31, 2015 to allow for a 12-month covariate ascertainment period prior to THA/TKA procedures performed in 2010, the first calendar year in which individuals were eligible to enter the cohort. Additionally, we utilized data from the Dartmouth Atlas Hospital Tracking File and Prescription Drug Abuse Policy System. The Prescription Drug Abuse Policy System summarized legislation relevant to state prescription drug monitoring programs effective through July 1, 2016, such as circumstances where prescribers are required to check the prescription drug monitoring programs [11]. Brown University’s institutional review board approved the study (protocol# 2010002823). Due to the use of deidentified administrative data, the need for informed consent was waived.

### Study participants

We designed this observational study to emulate a target trial comparing the safety of tramadol, hydrocodone, and oxycodone among U.S. Medicare beneficiaries with THA/TKA (Additional Table 1) [12]. Our study population consisted of Medicare Fee-for-Service beneficiaries aged 65 years or older on the date of elective inpatient THA/TKA procedure occurring between January 1, 2010 to September 30, 2015. THA/TKA procedures were identified based on Medicare Severity Diagnosis Related Groups codes 469 and 470 using inpatient claims. Eligible older adults were required to have 12 months of continuous Parts A and B enrollment, 6 months of continuous Part D enrollment, no opioid use in the 6 months immediately prior to the THA/TKA (Additional Table 2) [13], and no history of opioid-related inpatient hospitalizations or emergency department (ED) visits in the 12 months prior to THA/TKA. Individuals were excluded if they had Health Maintenance Organization (HMO) enrollment (Medicare Advantage) at any point prior to or during THA/TKA because these individuals' hospitalizations are not consistently reported by all hospitals [14]. We also excluded individuals with non-elective procedures, non-arthroplasty procedures, those admitted with fractures, those who were admitted for THA/TKA from a setting other than the community, and those utilizing hospice in the 12 months immediately prior to THA/TKA (Additional Table 3). Additionally, individuals initiating multiple opioids on the same day following THA/TKA (e.g., tramadol and hydrocodone); initiating opioids other than oxycodone, hydrocodone, or tramadol; or initiating opioids after seven days from THA/TKA were excluded. Lastly, we excluded beneficiaries discharged to post-acute care settings other than home with or without home health because medication dispensings through Part D cannot be observed [15]. For individuals with multiple procedures during the study period, only the first THA or TKA was included to better isolate initial opioid use.

### Opioid exposure

The interventions of interest were initiation of single opioid therapy with oxycodone, hydrocodone, or tramadol within seven days following THA/TKA hospital discharge regardless of concurrently administered nonopioid analgesics or pain adjuvants (e.g., acetaminophen, nonsteroidal anti-inflammatory drugs, gabapentinoids).

### Outcomes

We examined the first occurrence of two outcomes in the 90 days following THA/TKA: 1) all-cause hospitalizations and ED visits (all-cause serious adverse events [SAEs]) and 2) hospitalization and ED visits for a composite of 10 surgical- and opioid-related serious adverse events (composite SAEs) including THA/TKA revision, surgical complications [16, 17], cardiovascular events [18–21], fractures [22, 23], gastrointestinal events [24–26], acute liver injury [27], acute renal failure [28], opioid-related adverse events [29], delirium [30], and respiratory depression [31]. Outcomes were ascertained using *International Classification of Diseases, 9th Edition-Clinical Modification* (ICD-9-CM) diagnosis and procedure codes from MedPAR (inpatient hospital) and Part B Carrier (ED) claims (Additional Table 4).

### Follow-up

For each eligible participant, follow-up began at THA/TKA hospital discharge. Participants were followed until an outcome of interest or censoring event occurred. In an intention-to-treat (ITT) analysis, censoring events included death; disenrollment from Medicare Parts A, B, or D; HMO enrollment; or administrative end of follow-up (December 31, 2015 or day 90 of follow-up). Two additional censoring events were included in a per-protocol (PP) analysis: treatment discontinuation (the last available days’ supply based on a gap of no more than five days between refills) and treatment switching (initiation of a different opioid).

### Covariates

We measured 131 patient, hospital, and geographic characteristics prior to and concurrent with THA/TKA (Additional Table 5). We used the Medicare Master Beneficiary Summary File and Chronic Conditions Data Warehouse to ascertain beneficiary enrollment information, age, race/ethnicity [32], and medical conditions. Medication use in the 6 months prior to the THA/TKA was ascertained using Part D claims. MedPAR inpatient and Part B outpatient claims were used to calculate healthcare service utilization measures as well as the validated Combined Comorbidity Score and Claims-based Frailty Index [33, 34]. Hospital characteristics, including total beds, academic affiliation, and critical access hospital designation, were obtained from the Dartmouth Atlas Hospital Tracking file. Geographic characteristics included Department of Health and Human Services Region and “rigor” of the state prescription drug monitoring programs, which has been previously categorized using the Prescription Drug Abuse Policy System [11, 35].

### Causal contrasts

We were interested in both the ITT estimand (the effect of individuals being prescribed a treatment regardless of future adherence or treatment switching) and the PP estimand (the effect of individuals adhering to and remaining on the prescribed treatment) [36].

### Statistical analyses

The ITT estimand was estimated using stabilized inverse-probability-of-treatment-weighted (IPTW) pooled logistic regression models with robust error variance to adjust for potential confounding. The probabilities (i.e., propensity scores) used to construct the IPTWs were estimated via multinomial generalized boosted regression models [37, 38]. Estimated propensity scores showed substantial overlap (Additional Fig. 1). As an alternative approach, we estimated propensity scores using parametric multinomial logistic regression to assess if results differed when using non-parametric versus parametric approaches. For each subject, three probabilities were estimated, one for receiving each of the three treatments studied. The PP estimand was estimated using the same models as for the ITT estimand, but with the additional inclusion of inverse-probability-of-censoring-weights (IPCW) to reduce bias due to informative censoring. The probabilities of censoring used to construct the IPCW were estimated using logistic regression. We accounted for 131 covariates via IPTW and IPCW (Additional Table 5). Covariate balance was assessed using absolute standardized mean differences (ASMDs) with a difference of 0.1 or less considered to be indicative of sufficient covariate balance.

Pooled logistic regression models were employed for outcome estimation at the person-day level using the weighted pseudo-population after IPW [39]. Estimates were equivalent to those from discrete-time hazard models, which are interpretable as hazard ratios (HRs) with 95% confidence limits (CLs) (See Additional Appendix). The HRs provided a relative measure to compare the rates of outcomes among tramadol users versus oxycodone or hydrocodone users**.** We also estimated 90-day risk differences (RD) as an absolute measure using standardized survival curves [40]. Numbers needed to treat (NNT) and harm (NNH) were also estimated. Bootstrap standard errors (percentile method) for the RDs and NNTs/NNHs were estimated using 500 replications [41].

Additionally, a formal quantitative bias analysis was conducted by estimating E-values [42, 43].

Data were analyzed using SAS, version 9.4 (SAS Institute, Inc., Cary, NC), Stata, version 16 (StataCorp LLC, College Station, TX) and R, version 3.4 (R Foundation for Statistical Computing, Vienna, Austria).

## Results

### Study cohort

The study population included 28,769 opioid-naïve older adults with THA/TKA: 2,697 initiating tramadol, 11,407 initiating oxycodone, and 14,665 initiating hydrocodone (Additional Fig. 2, Table 1). Prior to IPW, tramadol users were older (75–84 years old 40.7% tramadol, 29.7% oxycodone, 34.1% hydrocodone), a greater proportion were female (70.1% tramadol, 53.8% oxycodone, 57.8% hydrocodone), had THA (34.9% tramadol, 28.2% oxycodone, 27.8% hydrocodone), heart failure (16.8% tramadol, 12.9% oxycodone, 13.8% hydrocodone), and were frailer (33.1% tramadol, 27.9% oxycodone, 29.5% hydrocodone) (Table 1). Time to opioid initiation after THA/TKA and censoring events were similar across treatment groups (Additional Table 6–7). Multiple covariates differed at baseline based on ASMDs of > 0.1, but were well balanced across treatment groups after applying IPW (Additional Table 8). We truncated the product of the IPTW and IPCW to the 99th percentile due to the presence of some extreme IPCW (Additional Table 9). However, the product IPW distributions did not differ markedly when propensity scores were estimated using generalized boosted regression or multinomial logistic regression models.

**Table 1:** Characteristics of Medicare beneficiaries initiating tramadol, oxycodone, or hydrocodone after total hip and knee arthroplasty

Characteristic	Tramadol (*n* = 2,697)	Oxycodone (*n* = 11,407)	Hydrocodone (*n* = 14,665)
Age, y, n (%)			
65–74	1,414 (52.4)	7,709 (67.6)	9,143 (62.3)
75–84	1,097 (40.7)	3,389 (29.7)	5,007 (34.1)
85 +	186 (6.9)	309 (2.7)	515 (3.5)
Female	1,890 (70.1)	6,142 (53.8)	8,470 (57.8)
Race/ethnicity, n (%)			
Non-Hispanic White	2,508 (93.0)	10,349 (90.7)	13,423 (91.5)
Non-Hispanic Black	56 (2.1)	413 (3.6)	363 (2.5)
Hispanic	69 (2.6)	283 (2.5)	512 (3.5)
Other	64 (2.4)	362 (3.2)	367 (2.5)
Dual-eligible, n (%)	136 (5.0)	699 (6.1)	974 (6.6)
THA, n (%)	940 (34.9)	3,216 (28.2)	4,076 (27.8)
TKA, n (%)	1,757 (65.1)	8,191 (71.8)	10,589 (72.2)
Discharge disposition, n (%)			
Home	1,002 (37.2)	3,538 (31.0)	5,446 (37.1)
Home with HH	1,695 (62.8)	7,869 (69.0)	9,219 (62.9)
Comorbidities, n (%)			
Alcohol use disorder	27 (1.0)	145 (1.3)	133 (0.9)
Tobacco use	97 (3.6)	545 (4.8)	674 (4.6)
Cancer	394 (14.6)	1,600 (14.0)	1,907 (13.0)
Dementia	109 (4.0)	302 (2.6)	451 (3.1)
AMI	75 (2.8)	266 (2.3)	334 (2.3)
AFib	324 (12.0)	1,204 (10.6)	1,471 (10.0)
HF	452 (16.8)	1,475 (12.9)	2,022 (13.8)
Hypertension	2,172 (80.5)	9,180 (80.5)	11,779 (80.3)
Ischemic Heart Disease	1,127 (41.8)	4,428 (38.8)	5,840 (39.8)
Stroke/TIA	239 (8.9)	759 (6.7)	1,053 (7.2)
Liver disease	147 (5.5)	548 (4.8)	681 (4.6)
CKD	396 (14.7)	1,514 (13.3)	1,776 (12.1)
Asthma	308 (11.4)	1,132 (9.9)	1,399 (9.5)
COPD	417 (15.5)	1,565 (13.7)	2,118 (14.4)
Combined Comorbidity Score, n (%)			
< 0^a^	531 (19.7)	2,619 (23.0)	3,402 (23.2)
0	764 (28.3)	3,436 (30.1)	4,541 (31.0)
1	588 (21.8)	2,324 (20.4)	2,962 (20.2)
2	345 (12.8)	1,351 (11.8)	1,729 (11.8)
3 +	469 (17.4)	1,677 (14.7)	2,031 (13.8)
Claims-based Frailty Index,^b^ n (%)			
Robust	1,805 (66.9)	8,225 (72.1)	10,343 (70.5)
Prefrail, Mildly Frail, or Moderately-to-severely Frail^c^	892 (33.1)	3,182 (27.9)	4,322 (29.5)

*Abbreviations*: *THA* total hip arthroplasty, *TKA* total knee arthroplasty, *HH* home health, *AMI* acute myocardial infarction, *AFib* atrial fibrillation, *HF* heart failure, *TIA* transient ischemic attack, *CKD* chronic kidney disease, *COPD* chronic obstructive pulmonary disease

^a^Combined Comorbidity Score values that are less than 0 are possible due to a component value of -1 in the algorithm for HIV/AIDS and Hypertension

^b^Measured using the Claims-based Frailty Index and categorized as: < 0.15 (robust), 0.15–0.24 (prefrail), 0.25–0.34 (mildly frail), and ≥ 0.35 (moderately-to-severely frail)

^c^Categories combined to avoid violating the Centers for Medicare and Medicaid Services Cell Size Suppression Policy

### Overall outcomes

The incidence of all-cause SAEs was 14.9% for tramadol, 13.4% for oxycodone, and 12.7% for hydrocodone in the ITT analysis and 10.3%, 9.2%, and 8.7%, respectively in the PP analysis (Additional Table 10). The incidence of composite SAEs was similar in the ITT analysis (3.1% tramadol, 2.8% oxycodone, 2.8% hydrocodone) and PP analysis (2.0% tramadol, 1.5% oxycodone, 1.7% hydrocodone).

### Relative estimates of outcomes

**Fig. 1:**
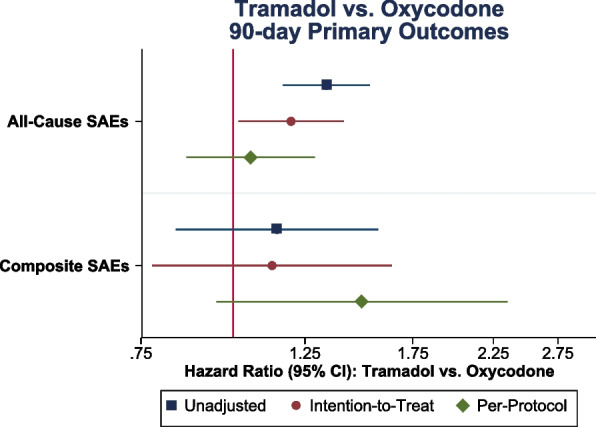
Crude and adjusted hazard ratios comparing postoperative tramadol versus oxycodone use for 90-day outcomes. Abbreviations: SAEs, Serious Adverse Events, SAEs; CI, Confidence Interval. Note: Outcome definitions for SAEs are listed in Additional Table 4. The unadjusted estimate is the intention-to-treat (ITT) estimand without covariate adjustment. Both the ITT and per-protocol estimates are covariate-adjusted

Initiating tramadol after THA/TKA was associated with increased rates of all-cause SAEs versus oxycodone based on the ITT estimand (HR 1.19, 95%CLs 1.02, 1.41) but no significant difference was observed based on the PP estimand (HR 1.05, 95%CLs 0.86, 1.29) (Fig. 1, Table 2). Rates of composite SAEs for tramadol versus oxycodone were not significantly different across comparisons (ITT HR 1.12, 95%CLs 0.78, 1.64; PP HR 1.49, 95%CLs 0.95, 2.35).

**Table 2:** Hazard ratios comparing postoperative opioid use for 90-day outcomes among older Medicare beneficiaries

Comparisons	90-day All-cause SAE	90-day Composite SAE
	**HR (95% CLs)**	**HR (95% CLs)**
**Tramadol vs Oxycodone (reference)**		
Unadjusted	1.34* (1.17, 1.53)	1.15 (0.84, 1.57)
Intention-to-Treat	1.19* (1.02, 1.41)	1.12 (0.78, 1.64)
Per-Protocol	1.05 (0.86, 1.29)	1.49 (0.95, 2.35)
**Tramadol vs Hydrocodone (reference)**		
Unadjusted	1.69* (1.38, 2.06)	1.15 (0.72, 1.83)
Intention-to-Treat	1.40* (1.10, 1.76)	1.19 (0.69, 2.04)
Per-Protocol	1.34* (1.03, 1.75)	1.63 (0.90, 2.95)

Notes: Average hazard ratio from pooled logistic regression model presented with 95% CL estimated using robust standard errors. Outcome definitions for SAEs are listed in Additional Table 4

*Abbreviations*: *ED* emergency department, *SAE* serious adverse events, *HR* hazard ratio, *CLs* confidence limits

^*^p-value < 0.05

**Fig. 2:**
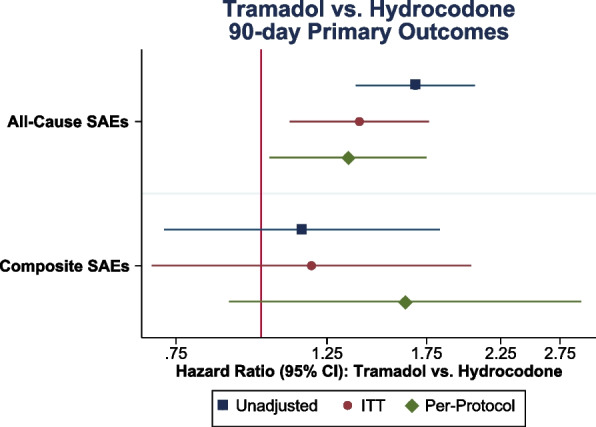
Crude and adjusted hazard ratios comparing postoperative tramadol versus hydrocodone use for 90-day outcomes. Abbreviations: SAEs, Serious Adverse Events, SAEs; CI, Confidence Interval. Note: Outcome definitions for SAEs are listed in Additional Table 4. The unadjusted estimate is the intention-to-treat (ITT) estimand without covariate adjustment. Both the ITT and per-protocol estimates are covariate-adjusted

Compared to hydrocodone, tramadol use was associated with increased rates of all-cause SAEs based on both the ITT (HR 1.40, 95% CLs 1.10, 1.76) and PP estimands (HR 1.34, 95%CLs 1.03, 1.75) (Fig. 2, Table 2). There were no significant differences in the rates of composite SAEs for tramadol versus hydrocodone (ITT HR 1.19, 95%CLs 0.69, 2.04; PP HR 1.63, 95%CLs 0.90, 2.95).

### Absolute estimates of outcomes

Tramadol initiators did not have markedly different 90-day RDs for all-cause SAEs or composite SAEs when compared to hydrocodone or oxycodone in the ITT and PP analyses (Table 3, Additional Fig. 3–8). Similarly, NNHs and NNTs were not significantly different for all-cause SAEs or composite SAEs in the ITT and PP analyses when tramadol was compared to oxycodone or hydrocodone (Additional Table 11).

**Table 3:** Risk differences comparing postoperative opioid use for 90-day outcomes among older Medicare beneficiaries

Comparisons	90-day All-cause SAE	90-day Composite SAE
	**RD, % (95% CLs)**	**RD, % (95% CLs)**
**Tramadol vs Oxycodone (reference)**		
Unadjusted	1.94* (0.31, 3.52)	0.35 (-0.41, 1.08)
Intention-to-Treat	0.78 (-1.00, 2.57)	0.14 (-0.66, 1.06)
Per-protocol	-0.49 (-5.66, 5.67)	3.01 (-0.48, 7.89)
**Tramadol vs Hydrocodone (reference)**		
Unadjusted	2.82* (1.32, 4.37)	0.30 (-0.40, 0.96)
Intention-to-Treat	1.11 (-0.64, 2.77)	0.05 (-0.71, 0.84)
Per-protocol	3.94 (-4.30, 11.61)	3.54 (-0.88, 9.03)

Notes: 90-day treatment-specific risks and risk differences were estimated from survival curves standardized to the joint distribution of covariates. The 95% CLs were estimated using the percentile-method from 500 bootstrap replications. Risks differences were multiplied by 100 and thus presented as percentage point differences. Outcome definitions for SAEs are listed in Additional Table 4

*Abbreviations*: *ED* emergency department, *SAE* serious adverse events, *RD* risk difference, *CL* confidence limits

^*^p < 0.05

### Quantitative bias analysis

Across all comparisons, E-values (lower CL) for all-cause SAEs ranged from 1.28 (1.00) to 2.77 (2.10) and 1.49 (1.00) to 2.64 (1.00) for composite SAEs (Additional Table 12).

## Discussion

In this nationally representative retrospective cohort study of older adults without opioid use in the 6 months prior to receiving elective THA/TKA, we found that tramadol does not have a superior safety profile compared to other opioids. Prescribing tramadol for acute postoperative pain was associated with an increased rate of all-cause SAEs compared to oxycodone or hydrocodone. Considering individuals who remained on the prescribed treatment (i.e., did not stop or switch opioids), tramadol was associated with an increased rate of all-cause SAEs versus hydrocodone only. However, there were no significant differences in the risk of all-cause SAEs based on absolute measures (i.e., RDs). No significant differences in the risk of composite SAEs were observed, possibly due to infrequent occurrence, limited statistical power, and imprecise estimates. The quantitative bias analyses suggested that a small to moderate amount of residual confounding could shift significant results to the null. Thus, interpreting results as causal effects may be imprudent.

Tramadol’s role in pain relief following THA/TKA has come under increased scrutiny, though limited guidance exists about how to optimally prescribe tramadol. Tramadol is mentioned in a 2020 guideline on the use of opioids following THA/TKA that was jointly developed by The American Association of Hip and Knee Surgeons, The American Academy of Orthopaedic Surgeons, The Hip Society, The Knee Society, and The American Society of Regional Anesthesia and Pain Medicine [9, 44]. The guideline explicitly acknowledges the limited evidence on both the postoperative adverse events and efficacy of oral tramadol, and calls for additional research. Some evidence suggests that tramadol is not more effective than other opioids [45, 46] and one study found that 11% of individuals receiving tramadol following TKA were converted to a stronger opioid due to inadequate pain relief [46]. Further, a 2020 consensus statement by the Enhanced Recovery After Surgery society recommends the use of acetaminophen and nonsteroidal anti-inflammatory drugs (NSAIDs), yet provides no guidance on tramadol use [1]. Thus, while recommendations have been made for opioid-sparing or opioid-free postoperative analgesia, guidance for treatment selection remains sparse. Such guidance is particularly important given older adults’ susceptibility to adverse events and the fact that they comprise a large, and growing, portion of the THA/TKAs performed each year. Thus, tramadol’s role in pain management among older adults requires a more nuanced evaluation.

Due to the lack of randomized controlled trials evaluating the comparative safety of tramadol relative to other opioids for acute postoperative pain, the evidence base for treatment decision-making is formed primarily from a few trials focused on chronic pain and some limited observational studies [9, 47, 48]. A small body of evidence generated among patients with osteoarthritis suggests worse outcomes, such as increased risk of all-cause mortality and hip fractures, with tramadol use compared to other opioid analgesics [49–51]. Short-term clinical studies in older adults with osteoarthritis prior to THA/TKA have shown a reduction in side effects when comparing tramadol to other opioids, such as respiratory depression and somnolence, but no differences in dizziness or headaches [52, 53]. Much of what is known of tramadol’s comparative safety relative to opioids or NSAIDs derives from its use to treat chronic pain resulting from osteoarthritis prior to THA/TKA [9, 54]. These studies are limited in their ability to assess tramadol’s safety compared to other opioids for postoperative pain management due to differences in pain etiology (i.e., acute vs. chronic) and absence of postoperative processes (e.g., rehabilitation). To our knowledge, our study is among the first to evaluate the safety of tramadol relative to other opioids among opioid-naïve older adults following elective THA/TKA. This study offers initial guidance as more providers shift toward opioid-sparing postoperative pain regimens for older adults. Our findings suggest that tramadol does not have a superior safety profile compared to other opioids and should not be preferentially prescribed to opioid-naïve older adults following THA/TKA. Therefore, additional research is needed to identify the safest regimen following THA/TKA that leverages multimodal analgesia and limits opioid-related adverse effects.

### Limitations

We acknowledge several potential limitations. First, these results may not generalize to adults younger than 65 years, those without Medicare Fee-for-Service insurance, with a history of opioid use or opioid-related adverse events prior to THA/TKA, residing in long-term care prior to surgery, or discharged to institutional post-acute care after THA/TKA. Individuals who filled their post-surgical opioid prescription prior to the elective procedure would have been excluded from the study population. However, given the nature of our data, we were not able to ascertain the medication indication and could not definitively distinguish between opioids for post-surgical pain management versus another pain indication.

Second, the quantitative bias analysis revealed E-values less than 2.2 for adjusted ITT and PP estimands, suggesting that a small to moderate amount of residual confounding could shift significant estimates to the null. Future work could consider adjusting for additional potential confounders, such as baseline and post-baseline measures of pain, concurrent initiation of other medications (e.g., acetaminophen, non-steroidal anti-inflammatory drugs, gabapentinoids) to manage post-operative pain, patient preferences, and social support.

Third, our data are restricted to medications that were dispensed; patients may not have taken the medications as prescribed, which could have resulted in exposure misclassification. This may have led to the introduction of bias into the PP estimands if misclassification due to incomplete adherence was differential across treatment groups. Additionally, we truncated the product of the IPTW and IPCW at the 99th percentile due to the presence of some extreme IPCW. This approach reduces variance, but may not reduce bias [55, 56]. Although the means of the IPCW were close to one, the extreme weights may indicate some model misspecification or possible violations of positivity. Concerns about positivity violations are merited since we 1) included a large number of covariates in the models used to estimate the probabilities of censoring and 2) estimated the models in person-day-level discrete time data.

Fourth, we could not ascertain adverse events that did not require inpatient hospitalization or an emergency department visit, such as falls not resulting in serious injuries. Opioid-related adverse events were rare in our administrative claims data and could only be examined as a composite outcome along with surgical-related adverse events. Other potential risks of opioid therapy, such as unintentional progression to long-term use and diversion, were not evaluated in this study, but remain a particularly important area of future research [57]. It would be beneficial for future work to compare the safety of post-operative tramadol versus hydrocodone or oxycodone on opioid-related adverse events using a dataset with more detailed clinical information that is not restricted to hospital and emergency department claims.

Lastly, while immortal time bias might be a concern in this study, the time between hospital discharge and post-operative use of each of the opioids was highly similar and the amount of time was very small, so any misclassification of time and resulting bias is likely to be non-differential and minimal.

## Conclusions

Our study found that initiation of postoperative tramadol following THA/TKA was associated with increased rates of all-cause SAEs compared to oxycodone and hydrocodone. However, there was no difference in the risk of composite SAEs. Since tramadol appears to be no safer than other opioids, it should not be preferentially prescribed to older adults for postoperative pain.

### Supplementary Information

**Supplementary Material 1.**

## Data Availability

The data that support the findings of this study are available from the Centers for Medicare and Medicaid Services (CMS) but restrictions apply to the availability of these data, which were used under data use agreements for the current study, and so are not publicly available. However, other researchers can establish their own data use agreement and obtain the datasets employed through the Research Data Assistance Center (ResDAC, https://resdac.org/), a CMS contractor that provides free assistance to researchers interested in CMS data.

## Abbreviations

ASMDs: Absolute standardized mean differences
CLs: Confidence limits
ED: Emergency department
HMO: Health Maintenance Organization
HRs: Hazard ratios
ITT: Intention-to-treat
IPCW: Inverse-probability-of-censoring-weight
IPTW: Inverse-probability-of-treatment-weight
MedPAR: Medicare Provider Analysis and Review
NNH: Number needed to harm
NNT: Number needed to treat
NSAIDs: Nonsteroidal anti-inflammatory drugs
PP: Per-protocol
RDs: Risk differences
SAEs: Serious adverse events
SNRI: Serotonin–norepinephrine reuptake inhibitor
THA/TKA: Total hip and knee arthroplasty
