# Supplementary material for: Comparative safety of tramadol and other opioids following total hip and knee arthroplasty
Source: BMC Geriatr. 2024 Apr 5;24:319. doi: 10.1186/s12877-024-04933-2 (PMC10996118; doi:10.1186/s12877-024-04933-2)
Supplement: Supplementary file 1 — Supplementary Material 1. [file 12877_2024_4933_MOESM1_ESM.docx]

**ADDITIONAL FILE 1**

**Authors:** Elliott Bosco, PharmD, PhD; Melissa R. Riester, PharmD; Francesca L. Beaudoin, MD, PhD; Andrew J. Schoenfeld, MD, MSc; Stefan Gravenstein, MD, MPH; Vincent Mor, PhD; Andrew R. Zullo, PharmD, PhD

**Title:** Comparative safety of tramadol and other opioids following total hip and knee arthroplasty.

**SUPPLEMENTARY APPENDIX**

**Additional Appendix.** Statistical analysis details.

**SUPPLEMENTARY FIGURES**

**Additional Figure 1.** Propensity score distributions estimated using multinomial Generalized Boosted Models.

**Additional Figure 2.** Flow diagram of the study population.

**Additional Figure 3.** Unadjusted 90-day standardized survival curves for all-cause serious adverse events.

**Additional Figure 4.** Inverse probability of treatment weighted 90-day standardized survival curves for all-cause serious adverse events.

**Additional Figure 5.** Inverse probability of treatment and inverse probability of censoring product-weighted 90-day standardized survival curves for all-cause serious adverse events.

**Additional Figure 6.** Unadjusted 90-day standardized survival curves for composite serious adverse events.

**Additional Figure 7.** Inverse probability of treatment weighted 90-day standardized survival curves for composite serious adverse events.

**Additional Figure 8.** Inverse probability of treatment and inverse probability of censoring product-weighted 90-day standardized survival curves for composite serious adverse events.

**SUPPLEMENTARY TABLES**

**Additional Table 1.** Protocol of the hypothetical target trial emulated to estimate the effects of postoperative opioids on safety outcomes among U.S. Medicare beneficiaries with total hip and knee arthroplasty.

**Additional Table 2.** Opioid analgesics identified using Medicare Part D claims in the 6 months prior to inpatient total hip and knee arthroplasty.

**Additional Table 3.** Codes used for sample eligibility (inclusion and exclusion) criteria.

**Additional Table 4.** Primary outcome definitions.

**Additional Table 5.** List of covariates accounted for in the treatment probability and censoring probability estimation models.

**Additional Table 6.** Time (in days) to treatment initiation (from hospital discharge date to first observed dispensing of an opioid of interest) across exposure groups.

**Additional Table 7.** Censoring events other than outcomes across treatment groups.

**Additional Table 8.** Absolute standardized differences prior to and after inverse probability of treatment weighting.

**Additional Table 9.** Stabilized inverse probability of treatment and censoring weight distributions.

**Additional Table 10.** Observed 90-day follow-up times and outcomes stratified by postoperative treatment.

**Additional Table 11.** Numbers needed to treat and harm for 90-day primary outcomes comparing postoperative tramadol use to oxycodone and hydrocodone.

**Additional Table 12.** E-values for formal quantitative bias analyses.

**SUPPLEMENTARY APPENDIX**

**Additional Appendix.** Statistical analysis details.

The intention-to-treat (ITT) estimand was estimated using stabilized inverse-probability-of-treatment-weighted (IPTW) pooled logistic regression models with robust error variance. The probabilities of treatment (i.e., propensity scores) used to construct the IPTWs were estimated non-parametrically via multinomial generalized boosted regression models (GBM).^1,2^ GBMs rely on iteratively fitting regression trees to achieve covariate balance between treatment groups.^1^ The propensity score model was fit with a maximum of 5,000 trees, an interaction depth of three, and to minimize the mean absolute differences in covariates between opioid exposure groups. For each subject, three propensity scores were estimated for each of the three treatments ($e_{0i}$,$e_{1i}$, $e_{2i}$). Covariates included in the propensity score model are found in **Additional Table 5** and consisted of baseline covariates that were ascertained prior to hospital discharge. Stabilized IPTWs were estimated to approximate baseline randomization to each treatment following THA/TKA (**Equation 1**).

$$SWtx=\frac{Pr(A=a)}{Pr(A=a| L_{o})} (1)$$

The probabilities of censoring used to construct the inverse-probability-of-censoring-weights (IPCWs) were estimated using pooled logistic regression models (**Equation 2**). IPCW models were fit separately in each treatment arm because the confounding structure may vary across treatments. IPCW were re-estimated for each outcome. Final weights for the per-protocol (PP) analysis were obtained by taking the product of the IPTW and IPCW.

$$SWcens\left( k \right)=\prod_{k=0}^{t} \frac{Pr(C\left( k \right)=0)}{Pr(C\left( k \right)=0|\bar{C}\left( k-1 \right)=0, L_{o})} (2)$$

The pooled logistic regression models used for outcome estimation were equivalent to discrete-time hazard models at the person-day level and thus produced estimates that are interpretable as hazard ratios (HRs) with 95% confidence intervals. The HRs provided a relative measure to compare the rates of outcomes among tramadol users versus oxycodone or hydrocodone users (**Equation 3**)**.** We also included interaction terms between treatment and follow-up time to allow the effect of treatment to vary over follow-up. Robust standard errors were estimated to account for within beneficiary correlation of observations in the weighted analysis.

$${Outcome}_{ik}={{}_{0}+}_{1}*{Treat}_{ik}+{}_{2}*{{Day}_{k}}+{}_{3}*{{Day}_{k}}^{2}+$$

$${}_{4}*{{Day}_{k}*{Treat}_{ik}}+{}_{5}*{Treat}_{ik}*{{Day}_{k}}^{2} (3)$$

**SUPPLEMENTARY FIGURES**

**Additional Figure 1.** Propensity score distributions estimated using multinomial Generalized Boosted Models.

**
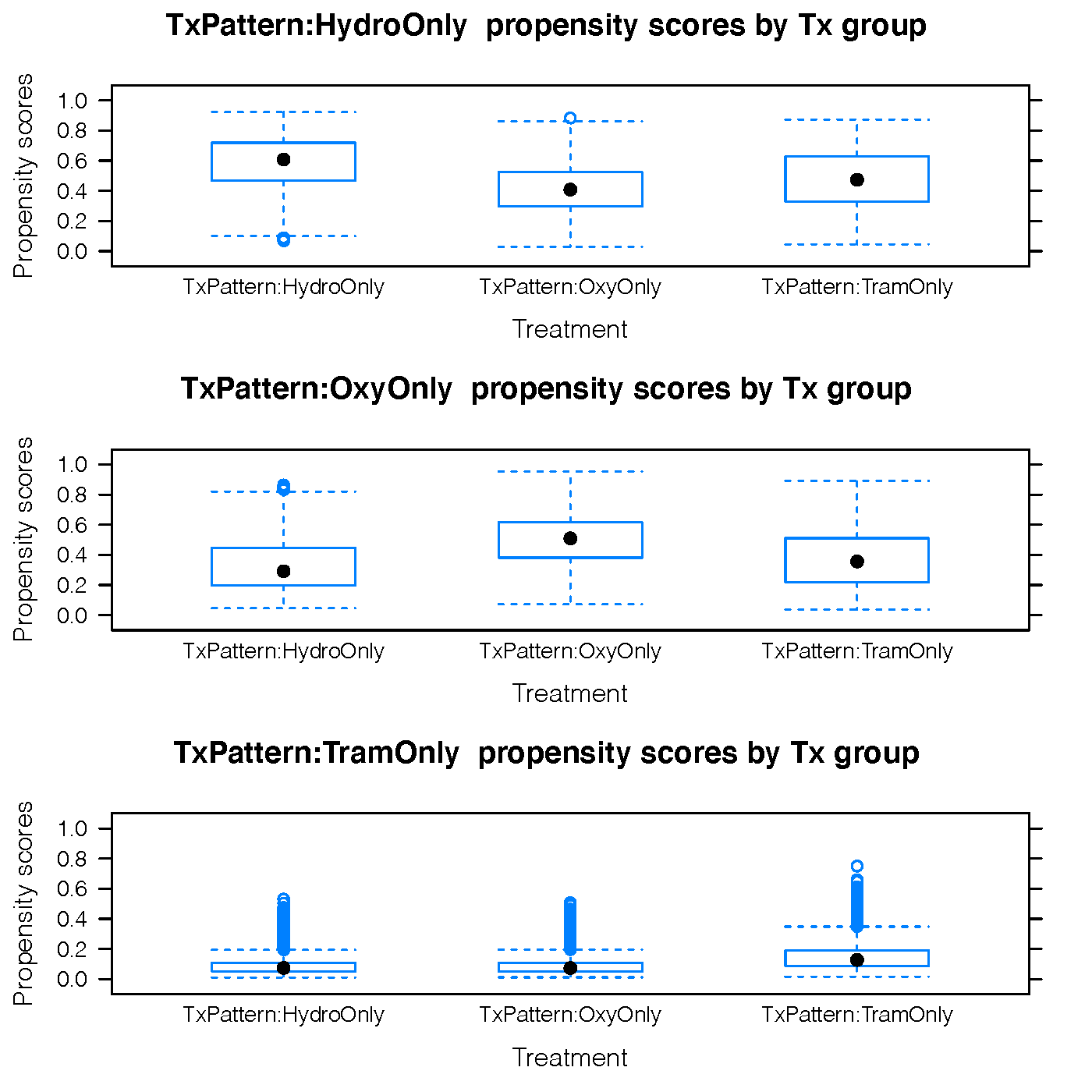
**

**Abbreviations**: HydroOnly, hydrocodone only; OxyOnly, oxycodone only; TramOnly, tramadol only; Tx, treatment; TxPattern, treatment pattern.

**Additional Figure 2.** Flow diagram of the study population.

**
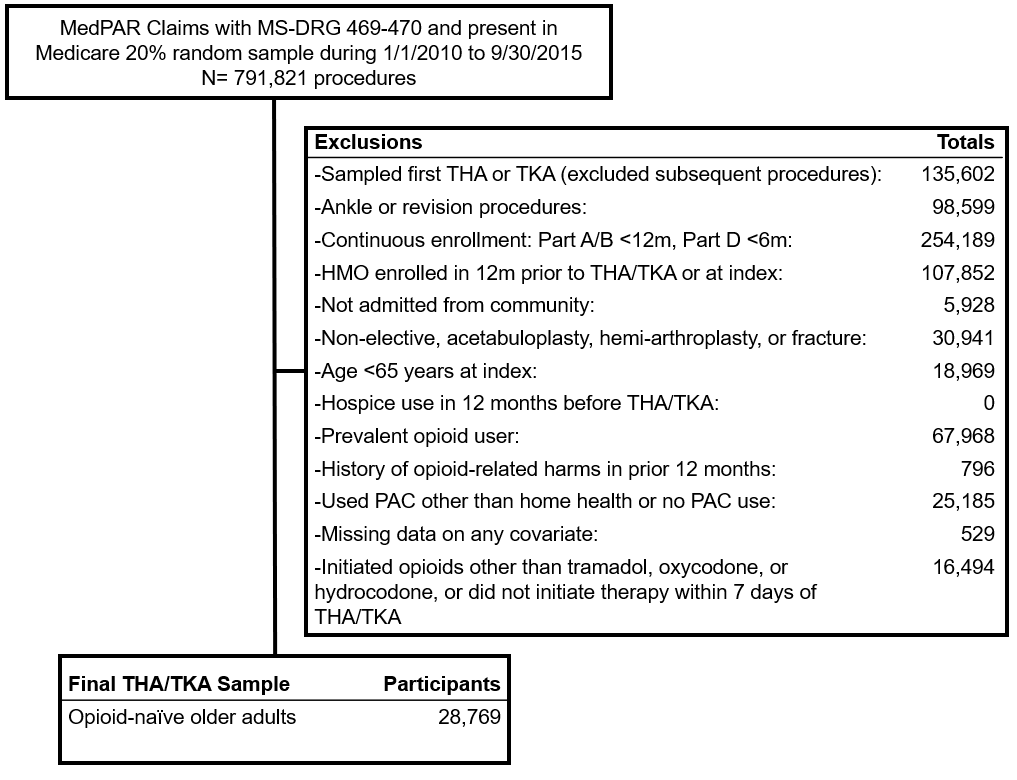
**

**Abbreviations:** MedPAR, Medicare Provider Analysis and Review; MS-DRG, Medicare Severity Diagnosis Related Groups; m, month; HMO, Health Maintenance Organization; THA/TKA, total hip and knee arthroplasty; PAC, post-acute care.

**Additional Figure 3.** Unadjusted 90-day standardized survival curves for all-cause serious adverse events.

**
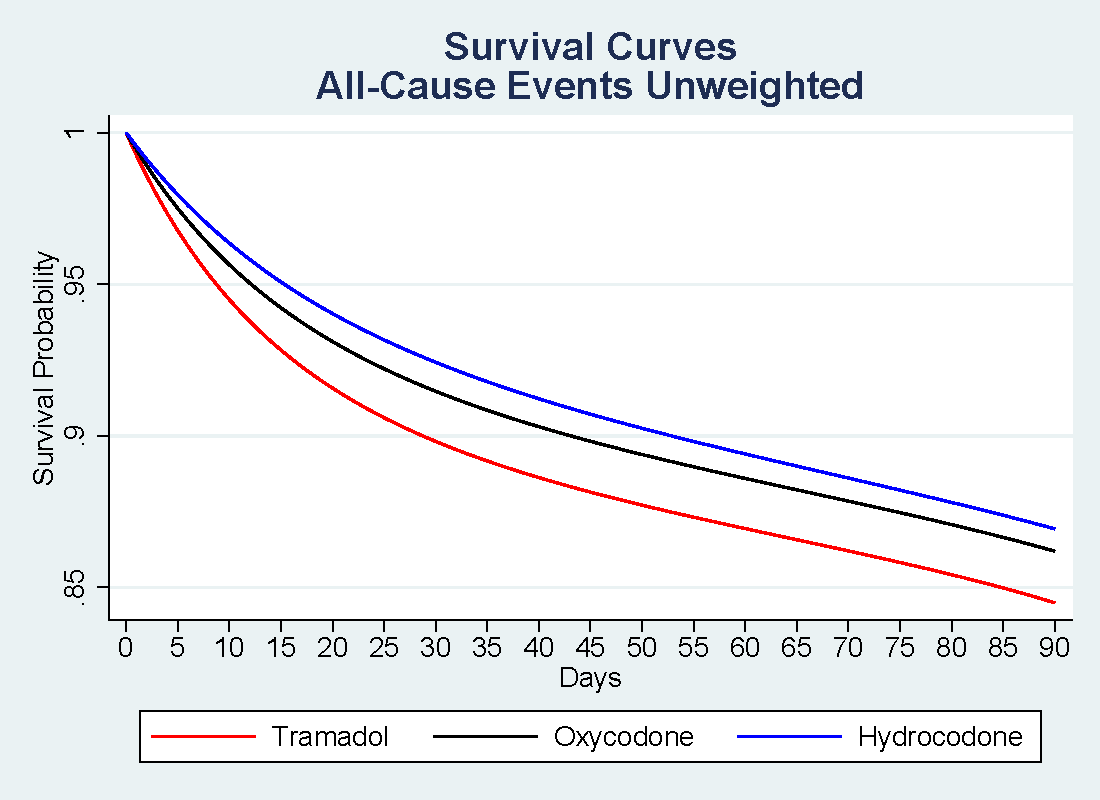
**

**Note:** Outcome definitions for serious adverse events are listed in Additional Table 4.

**Additional Figure 4.** Inverse probability of treatment weighted 90-day standardized survival curves for all-cause serious adverse events.

**
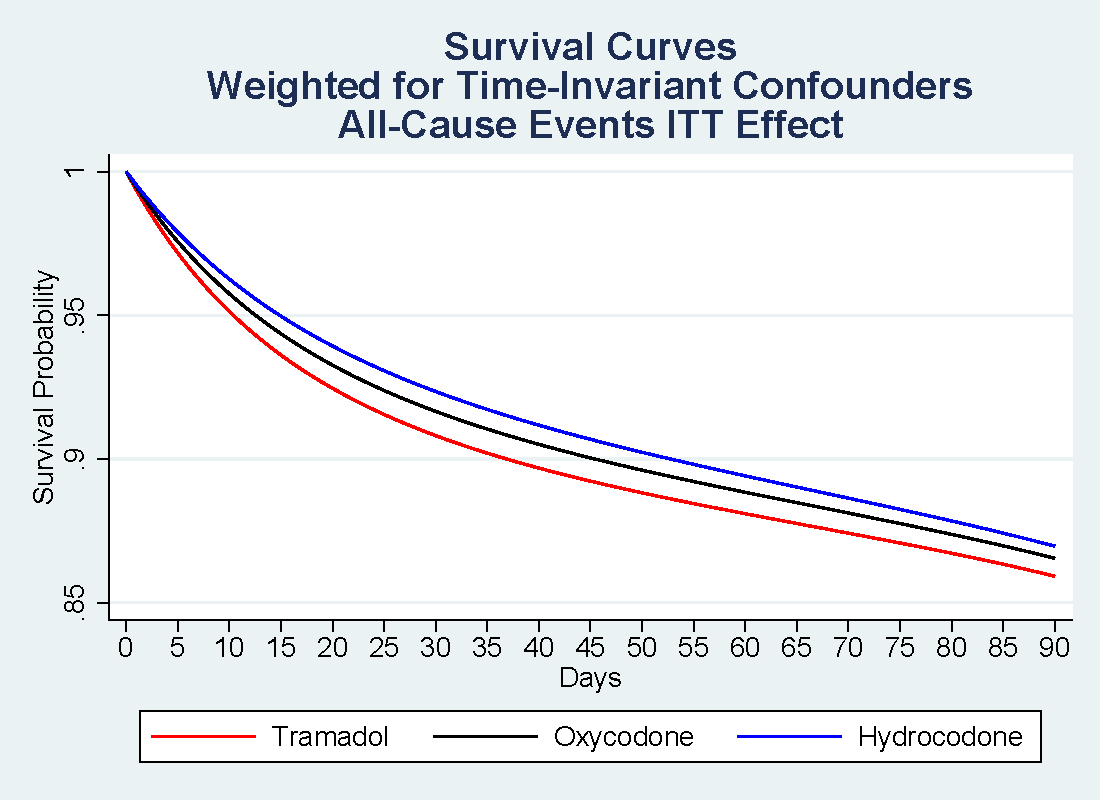
**

**Note:** Outcome definitions for serious adverse events are listed in Additional Table 4.

**Additional Figure 5.** Inverse probability of treatment and inverse probability of censoring product-weighted 90-day standardized survival curves for all-cause serious adverse events.

**
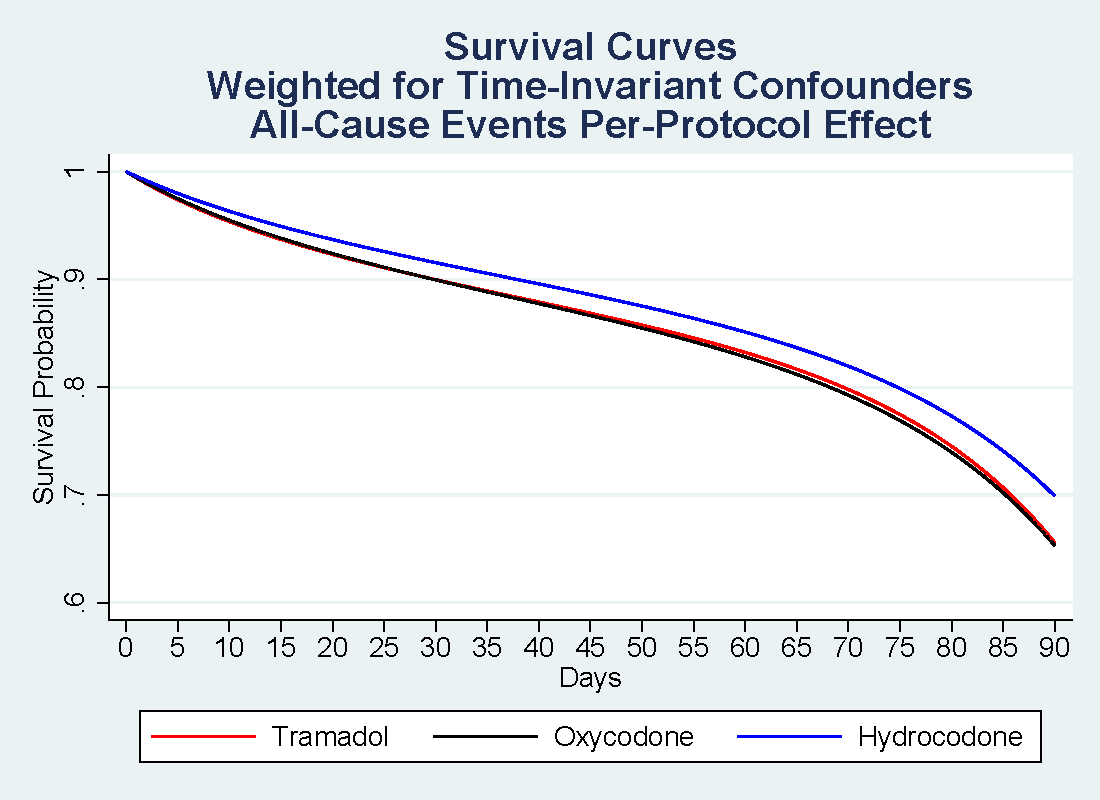
**

**Note:** Outcome definitions for serious adverse events are listed in Additional Table 4.

**Additional Figure 6.** Unadjusted 90-day standardized survival curves for composite serious adverse events.

**
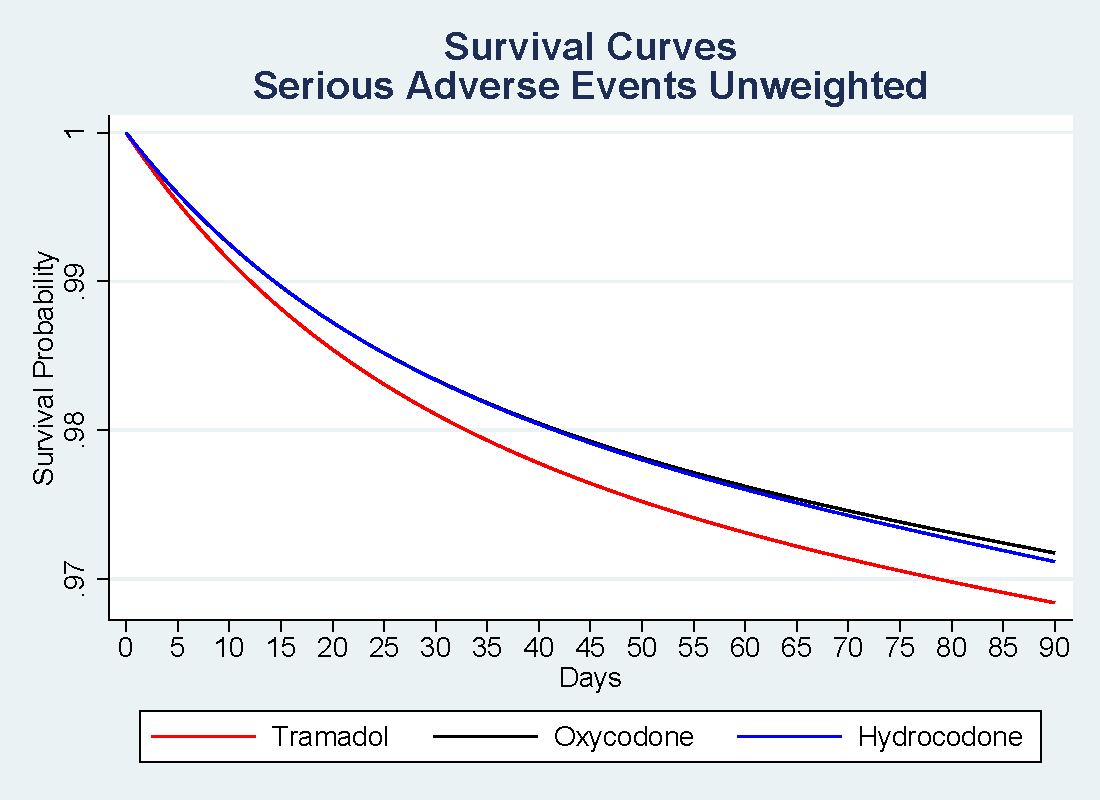
**

**Note:** Outcome definitions for serious adverse events are listed in Additional Table 4.

**Additional Figure 7.** Inverse probability of treatment weighted 90-day standardized survival curves for composite serious adverse events.

**
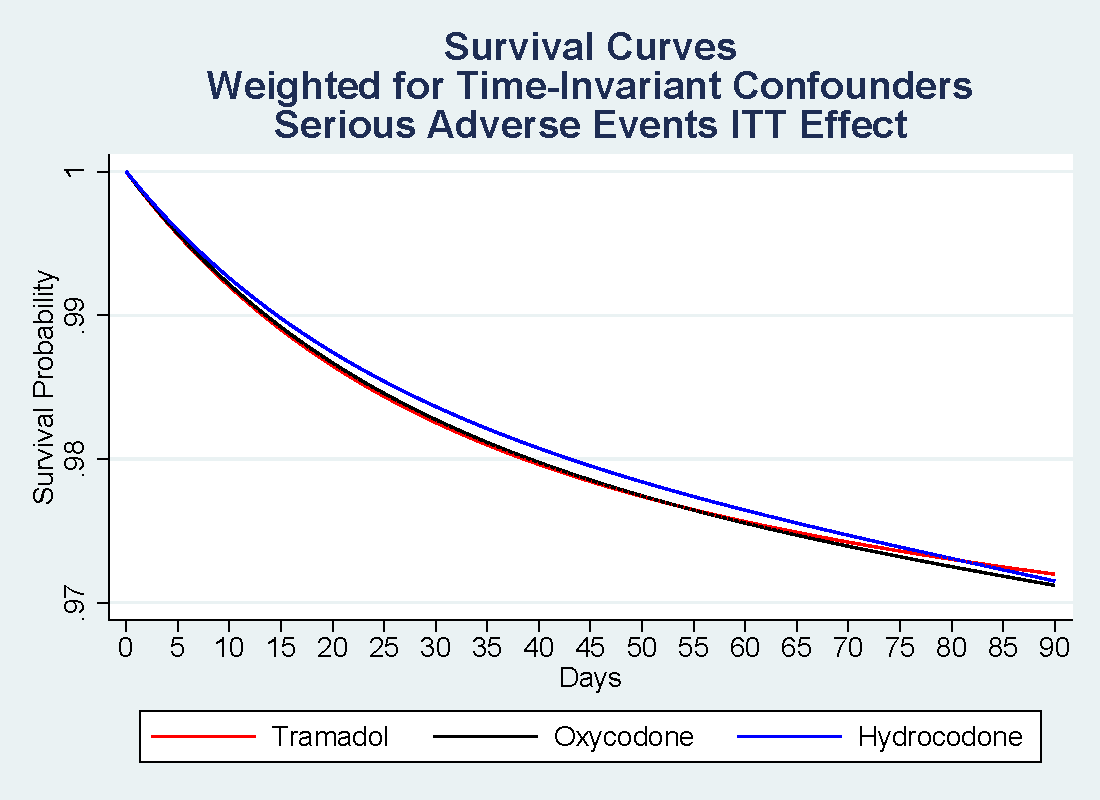
**

**Note:** Outcome definitions for serious adverse events are listed in Additional Table 4.

**Additional Figure 8.** Inverse probability of treatment and inverse probability of censoring product-weighted 90-day standardized survival curves for composite serious adverse events.

**
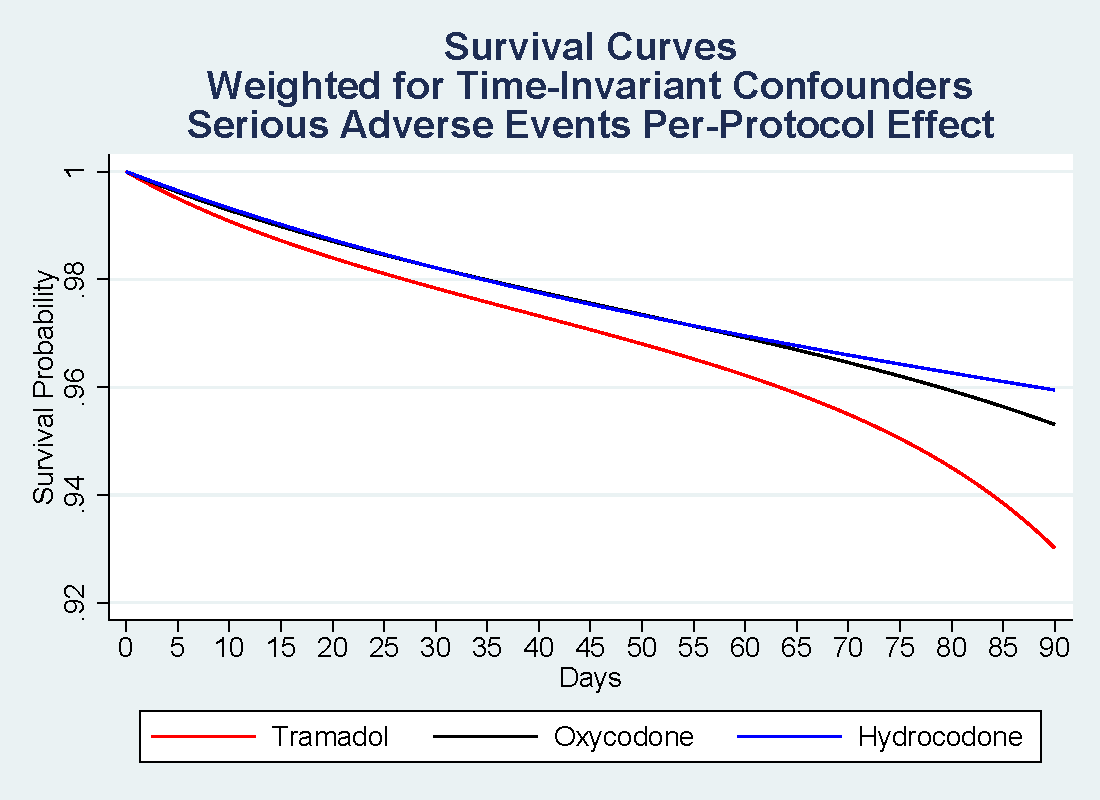
**

**Note:** Outcome definitions for serious adverse events are listed in Additional Table 4.

**SUPPLEMENTARY TABLES**

**Additional Table 1.** Protocol of the hypothetical target trial emulated to estimate the effects of postoperative opioids on safety outcomes among U.S. Medicare beneficiaries with total hip and knee arthroplasty.

| **Protocol Component** | **Description of Target Trial** | **Description of Emulation Using Medicare Data** |
| --- | --- | --- |
| Eligibility criteria | - Community-dwelling U.S. adults aged ≥65 years between January 1, 2010 to September 30, 2015. - Received their first elective total hip or knee arthroplasty (THA/TKA) and were discharged to the community. - Continuously enrolled in Medicare Parts A and B for 12 months and Part D for 6 months prior to THA/TKA. - Opioid-naïve (no opioid use in the 6 months prior to THA/TKA). - No history of opioid-related harms (e.g., opioid overdose) in the 12 months prior to THA/TKA. - No hospice in the 12 months prior to THA/TKA. | - Same as target trial, but the eligibility criteria were applied by searching for documentation in Medicare claims data (**Additional Table 3**). - We sampled only the first THA/TKA *during the study period*, but is possible that individuals had additional THA/TKAs performed before study period began or before they enrolled in the Medicare program. |
| Treatment strategies | 1. Tramadol: Patient initiates single opioid therapy (i.e., does not initiate multiple opioids on the same day) with any dose of tramadol at the time of discharge from the hospital for THA/TKA.  2. Oxycodone: Patient initiates single opioid therapy with any dose of oxycodone at the time of discharge from the hospital for THA/TKA.  3. Hydrocodone: Patient initiates single opioid therapy with any dose of hydrocodone at the time of discharge from the hospital for THA/TKA.  *Note:* Under any of the three treatment strategies, the decision to discontinue or initiate any additional therapies is left to the patient and their healthcare professionals’ discretion. Any use of concomitant nonopioid analgesics (e.g., acetaminophen, non-steroidal anti-inflammatory drugs, gabapentinoids) is acceptable | - Same as target trial, but treatment was ascertained using the Medicare Part D prescription drug dispensing claims. - Individuals were classified according to the strategy with which their data were compatible within the seven days following THA/TKA hospital discharge. - Identifying treatment strategies exactly at hospital discharge is often impossible since individuals are provided a limited supply of medication by the hospital and this limited supply is unobservable in Part D claims data (because it is bundled into the hospital payment); identifying treatment strategies within seven days of hospital discharge represents a deviation from the target trial. |
| Treatment assignment procedures | - Participants were randomized to a treatment strategy at baseline (the time of hospital discharge) and they and their clinicians were aware of the assigned strategy. | - Individuals (non-randomly) assigned by their clinicians to each opioid treatment strategy are assumed to be comparable conditional on the 131 baseline covariates listed in **Additional Table 5** (i.e., assignment is assumed to be random within covariate strata). |
| Follow-up period | - Intention-to-treat: For each eligible person, follow-up started when the person was assigned to a treatment strategy (time zero) and ended at the earliest of disenrollment from Medicare fee-for-service (from Parts A, B, or D) or enrollment in health maintenance organization (Medicare Advantage), death, an outcome event, 90 day of follow-up, or study end (December 31, 2015). - Per-protocol: Same as intention-to-treat above, but two additional criteria are considered: treatment discontinuation and treatment switching. | - Same as the target trial, but start of follow-up (time zero) is at discharge from the THA/TKA procedure hospitalization rather than assignment to treatment. - For the per-protocol analysis, treatment discontinuation is identified as the last available days’ supply of a prescription with a gap of no more than five days between refills; treatment switching is identified as initiating a different opioid-containing agent. |
| Outcomes | - Hospitalization or emergency department visit for:   1) Any (all) cause.  2) A surgical- or opioid-related serious adverse event (THA/TKA revision, surgical complications, cardiovascular events, fractures, gastrointestinal events, acute liver injury, acute renal failure, opioid-related adverse events, delirium, or respiratory depression). | - Same as target trial; outcomes are identified using combinations of International Classification of Diseases, 9^th^ Revision, Clinical Modification (ICD-9), Current Procedural Terminology (CPT), and Healthcare Common Procedure Coding System (HCPCS) codes identified in the Medicare Parts A and B claims (**Additional** **Table 4**). |
| Causal contrasts | - Intention-to-treat effect.   - The effect of being assigned to any dose of tramadol at baseline versus either: i) any dose of oxycodone at baseline, or ii) any dose of hydrocodone at baseline regardless of whether participants adhered to their assigned treatment strategy during follow-up (i.e., regardless of treatment discontinuation or switching). - Per-protocol effect.   - The effect of adhering to any dose of tramadol throughout follow-up versus either: i) adhering to any dose of oxycodone, or ii) adhering to any dose of hydrocodone. Participants are censored for treatment discontinuation or switching opioid treatment strategy. | - Same as the target trial, but both estimands are the observational study analogs. |
| Statistical analysis | - Intention-to-treat: Estimate hazard ratios comparing the treatment groups with treatment as the only covariate using a pooled logistic regression model. - Per-protocol: Same as intention-to-treat above, but individuals are censored when their data stop being compatible with the strategy they are assigned to and each individual receives an inverse probability of censoring weight to adjust for the potential selection bias introduced by censoring. | - Same as the target trial, but inverse probability weights must be estimated.   - Intention-to-treat: Pooled logistic regression model to correct for potential confounding between treatment groups using the inverse probability of treatment weights accounting for 131 baseline covariates.   - Per-protocol: Same as intention-to-treat above, but also use estimated inverse probability of censoring weights to correct for potential differential (informative) censoring between treatment groups using 131 baseline covariates. |

**Additional Table 2.** Opioid analgesics identified using Medicare Part D claims in the 6 months prior to inpatient total hip and knee arthroplasty.

| **Opioid Analgesics** |
| --- |
| Hydrocodone  Oxycodone  Hydromorphone  Oxymorphone  Methadone  Codeine  Meperidine  Morphine  Fentanyl  Pentazocine  Tapentadol  Tramadol |

**Additional Table 3.** Codes used for sample eligibility (inclusion and exclusion) criteria.

| **Inclusion/Exclusion** | **Criteria** | **Code Type** | **Codes Used** |
| --- | --- | --- | --- |
| Inclusion | THA/TKA performed | MS-DRG  and  ICD-9-CM Procedures derived from inpatient claims (in any coding position) | 469-470  and  8151 (total hip), 8154 (total knee) |
| Exclusion | Non-elective, acetabuloplasty, hemi-arthroplasty, ankle and revision procedures, and fractures | CPT documented in Part B claims (in any coding position)  or  ICD-9-CM Diagnoses documented in inpatient claims (in principal coding position)  or  ICD-9-CM Procedures documented in inpatient claims (in any coding position) | Acetabuloplasty and hemi-arthroplasty CPT: 27120, 27122, 27125  or  Fractures ICD-9-CM, starting with: 820  or  8152 (partial hip), 8156 (ankle) |

**Abbreviations:** THA/TKA, total hip and knee arthroplasty; MS-DRG, Medicare Severity Diagnosis Related Group; CPT, Current Procedural Terminology; ICD-9-CM, International Classification of Diseases, 9^th^ Revision, Clinical Modification.

**Additional Table 4.** Primary outcome definitions.

| **Outcome/Grouping** | **Description** | **Codes with validation citation** |
| --- | --- | --- |
| Composite Outcomes | | |
| All-cause hospitalizations and ED visits (all-cause serious adverse events) | | N/A |
| Hospitalizations and ED visits for serious surgical- and opioid-related adverse events (composite serious adverse events) | | Composed of specific serious adverse event endpoints below (identified in any coding position). |
| Specific Serious Adverse Events | | |
| THA/TKA revision operations | | ICD-9-Procedure: 8153, 8155  CPT: 27134, 27137, 27138, 27486, 27487 |
| Surgical complications | Broad bacterial infections and surgical site infections (SSI). | Broad bacterial infections^3^  SSI^4^  ICD-9-CM: 320, 049, 323, 681, 682, 421, 481, 482, 590, 038, 0543, 7907, 6110, 6830, 6113, 8750, 8751, 8790, 8791, 6822, 6823, 6829, 9983, 9986, 71100, 71101, 71102, 71103, 71104, 71105, 71106, 71107, 71109, 73000, 73001, 73002, 73003, 73004, 73005, 73006, 73007, 73008, 73009, 73010, 73011, 73012, 73013, 73014, 73015, 73016, 73017, 73018, 73019, 73020, 73021, 73022, 73023, 73024, 73025, 73026, 73027, 73028, 73029, 99669, 99850, 99851, 99852, 99853, 99854, 99855, 99856, 99857, 99858, 99859, 04110, 04111, 04112, 04113, 04114, 04115, 04116, 04117, 04118, 04119, 56782, 99812, 99832, 99883  ICD-9-Procedure: 850, 540, 543, 8591, 8594, 8596, 8344, 8345, 8346, 8347, 8348, 8349, 8601, 8604, 8609, 8622, 8628 |
| Cardiovascular events | Acute myocardial infarction (AMI),  acute ischemic stroke (AIS),  heart failure (HF)  sudden cardiac death (SCD) | Validated algorithms (PPV of ≥86%) employing ICD-9 codes in primary position for  AMI (ICD-9-CM: 410.x0; 410.x1),^5^  AIS (ICD-9-CM: 433.x1; 434.x1; 436.x),^6^  HF (ICD-9-CM: 428.X)^7^  Sudden cardiac death (death from enrollment file and ICD-9-CM: 427.5; 798; 798.1; 798.2)^8^  ICD-9-CM Codes for SCD + death: 4275; 798; 7981; 7982  ICD-9-CM: 436, 428, 41000, 41010, 41020, 41030, 41040, 41050, 41060, 41070, 41080, 41090, 41001, 41011, 41021, 41031, 41041, 41051, 41061, 41071, 41081, 41091, 43301, 43311, 43321, 43331, 43341, 43351, 43361, 43371, 43381, 43391, 43401, 43411, 43421, 43431, 43441, 43451, 43461, 43471, 43481, 43491 |
| Fractures | Vertebral and nonvertebral (hip, wrist, pelvis, humerus) | Vertebral compression fracture^9^  Non-vertebral fracture^10^  ICD-9 E-codes: E880, E881, E882, E883, E884, E885, E886, E887, E888, E9293  ICD-9-CM: 73313, 820, 813, 812, 808, 824, 821, 814, 815, 816, 817, 823, 800, 801, 802, 803, 804, 825, 826, 810, 811, 822, 809, 818, 819, 827, 828, 829, 8052, 8054, 8058, 8070, 8071, 8072, 8073, 8074, 8056, 8057, 8066, 8067 |
| Gastrointestinal (GI) events | Upper and lower GI bleeds, bowel obstruction | Peptic ulcers (ICD-9-CM: 531, 532, 533), Gastritis with hemorrhage (535.01, 535.41, 535.51), gastrointestinal hemorrhage (578)^11^  Upper GI bleed^12^  Bowel obstruction: No validation of algorithm.^13^  ICD-9-CM: 531, 532, 533, 578, 5310, 5311, 5312, 5313, 5314, 5315, 5316, 5317, 5319, 5320, 5321, 5322, 5323, 5324, 5325, 5326, 5327, 5329, 5330, 5331, 5332, 5333, 5334, 5335, 5336, 5337, 5339, 5340, 5341, 5342, 5343, 5344, 5345, 5346, 5347, 5349, 5780, 5781, 5789, 5609, 53501, 53541, 53551, 56081, 56089 |
| Severe acute liver injury | | Acute liver injury^14^  ICD-9-CM: 570, 5724. |
| Acute renal failure | | Acute renal failure^15^  ICD-9-CM: 5845, 5846, 5847, 5848, 5849 |
| Opioid-related adverse events | | ICD-9-CM codes for opioid-related harms derived from AHRQ^16,17^  ICD-9-CM: 30550, 30551, 30552, 30400, 30401, 30402, 30470, 30471, 30472, 96500, 96501, 96502, 96509, 9701  ICD-9-CM E-codes: E9350, E9351, E9352, E9401, E8500, E8501, E8502 |
| Delirium | | Delirium^18^  ICD-9-CM: 2930, 2931, 2903, 2910, 2939, 2930, 2931, 2903, 2910, 2939, 2922, 2902, 2908, 2909, 2920, 3483, 29281, 29011, 29041, 78009, 29381, 29382, 29383, 29384, 29389, 29281, 29011, 29041, 78009, 29381, 29382, 29383, 29384, 29389, 29012, 29013, 29043, 29211, 29212, 78002, 29042, 29282, 34831, 34839, 34982, 78097 |
| Respiratory depression | | Respiratory Depression^19^  ICD-9-CM: 5188 or 51881 |

**Abbreviations:** THA/TKA, total hip and knee arthroplasty; ICD-9-CM, International Classification of Diseases, 9^th^ Revision, Clinical Modification; CPT, Current Procedural Terminology; PPV, positive predictive value; E-codes, external cause of injury codes; HCPCS, Healthcare Common Procedure Coding System.

**Additional Table 5.** List of covariates accounted for in the treatment probability and censoring probability estimation models.

| **Variable** | **Description** | **Values** |
| --- | --- | --- |
| **Patient Characteristics** | | |
| thatkayear | Year of THA/TKA | 2010, 2011, 2012, 2013, 2014, 2015 |
| pxqrtr | Quarter of year of THA/TKA | January-March, April-June, July-September, October-December |
| tramadoldea | THA/TKA occurred after DEA scheduling of tramadol from unscheduled to Schedule IV (July 2014) | 0=before, 1=after |
| hydrodea | THA/TKA occurred after DEA scheduling of hydrocodone from Schedule III to Schedule II (October 2014) | 0=before, 1=after |
| loscat | Length of stay THA/TKA category | 1 day, 2 days, 3+days |
| hksex | Sex | 0=male, 1=female |
| nagecat | Age category | 65-74, 75-84, 85+ |
| nrace | Race/ethnicity | White non-Hispanic; Black non-Hispanic; Hispanic; Other |
| nhipknee | THA or TKA performed | THA, TKA |
| dualany12pre | Any dual eligibility in the 12 months prior to THA/TKA | 0=no, 1=yes |
| pre180benzo_Max | Prior use (180 days) of benzodiazepines | 0=absent, 1=present |
| pre180cox2_Max | Prior use (180 days) of COX-2 inhibitors | 0=absent, 1=present |
| pre180semicox_Max | Prior use (180 days) of semi-selective COX inhibitors | 0=absent, 1=present |
| pre180noncox_Max | Prior use (180 days) of nonselective COX inhibitors | 0=absent, 1=present |
| pre180apap_Max | Prior use (180 days) of acetaminophen | 0=absent, 1=present |
| pre180irrevcox_Max | Prior use (180 days) of irreversible COX inhibitor | 0=absent, 1=present |
| pre180ppi_Max | Prior use (180 days) of proton pump inhibitor | 0=absent, 1=present |
| pre180gabas_Max | Prior use (180 days) of gabapentinoids | 0=absent, 1=present |
| pre180anticonv_Max | Prior use (180 days) of anticonvulsants | 0=absent, 1=present |
| pre180musrelax_Max | Prior use (180 days) of muscle relaxants | 0=absent, 1=present |
| pre180abx_Max | Prior use (180 days) of antibiotics | 0=absent, 1=present |
| pre180ace_Max | Prior use (180 days) of ACE-inhibitors | 0=absent, 1=present |
| pre180antiplate_Max | Prior use (180 days) of antiplatelets | 0=absent, 1=present |
| pre180antixa_Max | Prior use (180 days) of anti-Xa inhibitors | 0=absent, 1=present |
| pre180arb_Max | Prior use (180 days) of ARBs | 0=absent, 1=present |
| pre180beta_Max | Prior use (180 days) of beta-blockers | 0=absent, 1=present |
| pre180cortico_Max | Prior use (180 days) of corticosteroids | 0=absent, 1=present |
| pre180dhccb_Max | Prior use (180 days) of dihydropyridine CCBs | 0=absent, 1=present |
| pre180nondhccb_Max | Prior use (180 days) of nondihydropyridine CCBs | 0=absent, 1=present |
| pre180hypnotics_Max | Prior use (180 days) of non-benzodiazepine hypnotics | 0=absent, 1=present |
| pre180insulin_Max | Prior use (180 days) of insulin | 0=absent, 1=present |
| pre180noninsdiab_Max | Prior use (180 days) of non-insulin diabetes medications | 0=absent, 1=present |
| pre180ksparing_Max | Prior use (180 days) of potassium-sparing diuretics | 0=absent, 1=present |
| pre180loop_Max | Prior use (180 days) of loop diuretics | 0=absent, 1=present |
| pre180maoi_Max | Prior use (180 days) of MAO-inhibitors | 0=absent, 1=present |
| pre180mirtaz_Max | Prior use (180 days) of mirtazapine | 0=absent, 1=present |
| pre180miso_Max | Prior use (180 days) of misoprostol | 0=absent, 1=present |
| pre180mixed5ht_Max | Prior use (180 days) of mixed 5-HT | 0=absent, 1=present |
| pre180ndri_Max | Prior use (180 days) of NDRIs | 0=absent, 1=present |
| pre180nitrate_Max | Prior use (180 days) of nitrates | 0=absent, 1=present |
| pre180osteopor_Max | Prior use (180 days) of osteoporosis medications | 0=absent, 1=present |
| pre180snri_Max | Prior use (180 days) of SNRIs | 0=absent, 1=present |
| pre180ssri_Max | Prior use (180 days) of SSRIs | 0=absent, 1=present |
| pre180statin_Max | Prior use (180 days) of statins | 0=absent, 1=present |
| pre180tca_Max | Prior use (180 days) of TCAs | 0=absent, 1=present |
| pre180thiazide_Max | Prior use (180 days) of thiazide diuretics | 0=absent, 1=present |
| pre180vaptan_Max | Prior use (180 days) of vaptans | 0=absent, 1=present |
| pre180warfarin_Max | Prior use (180 days) of warfarin | 0=absent, 1=present |
| numpriormed | Number of distinct medications used in prior 12 months. | 0 or more |
| nfrailtycat | Claims-based frailty index category | Robust, Prefrail, Mildly Frail, Moderately-to-severely Frail |
| ncomorbidcat | Combined comorbidity score category | <0, 0, 1, 2, 3+ |
| opabusehx | History of opioid-related adverse events | 0=absent, 1=present |
| cancerflag | History of cancer | 0=absent, 1=present |
| Alcohol_abuse | Comorbidity score condition: History of alcohol abuse | 0=absent, 1=present |
| ccw_acp | CCW condition: ADHD, Conduct Disorders, and Hyperkinetic Syndrome | 0=absent, 1=present |
| ccw_alz | CCW condition: Alzheimer's Disease | 0=absent, 1=present |
| ccw_alzdem | CCW condition: Alzheimer's Disease, Related Disorders, or Senile Dementia | 0=absent, 1=present |
| ccw_ami | CCW condition: Acute Myocardial Infarction | 0=absent, 1=present |
| ccw_anem | CCW condition: Anemia | 0=absent, 1=present |
| ccw_anxi | CCW condition: Anxiety Disorders | 0=absent, 1=present |
| ccw_arthr | CCW condition: Rheumatoid Arthritis / Osteoarthritis | 0=absent, 1=present |
| ccw_asth | CCW condition: Asthma | 0=absent, 1=present |
| ccw_atrfib | CCW condition: Atrial Fibrillation | 0=absent, 1=present |
| ccw_autism | CCW condition: Autism Spectrum Disorders | 0=absent, 1=present |
| ccw_bipl | CCW condition: Bipolar Disorder | 0=absent, 1=present |
| ccw_bph | CCW condition: Benign Prostatic Hyperplasia | 0=absent, 1=present |
| ccw_brainj | CCW condition: Brain Injury | 0=absent, 1=present |
| ccw_cabrst | CCW condition: Cancer, Breast | 0=absent, 1=present |
| ccw_cacolo | CCW condition: Cancer, Colorectal | 0=absent, 1=present |
| ccw_caendo | CCW condition: Cancer, Endometrial | 0=absent, 1=present |
| ccw_calung | CCW condition: Cancer, Lung | 0=absent, 1=present |
| ccw_capros | CCW condition: Cancer, Prostate | 0=absent, 1=present |
| ccw_catar | CCW condition: Cataract | 0=absent, 1=present |
| ccw_cerpal | CCW condition: Cerebral Palsy | 0=absent, 1=present |
| ccw_chf | CCW condition: Heart Failure | 0=absent, 1=present |
| ccw_chrkid | CCW condition: Chronic Kidney Disease | 0=absent, 1=present |
| ccw_copd | CCW condition: Chronic Obstructive Pulmonary Disease | 0=absent, 1=present |
| ccw_cysfib | CCW condition: Cystic Fibrosis and Other Metabolic Developmental Disorders | 0=absent, 1=present |
| ccw_depr | CCW condition: Depressive Disorders | 0=absent, 1=present |
| ccw_depsn | CCW condition: Depression | 0=absent, 1=present |
| ccw_diab | CCW condition: Diabetes | 0=absent, 1=present |
| ccw_epilep | CCW condition: Epilepsy | 0=absent, 1=present |
| ccw_fibro | CCW condition: Fibromyalgia, Chronic Pain and Fatigue | 0=absent, 1=present |
| ccw_glauc | CCW condition: Glaucoma | 0=absent, 1=present |
| ccw_hearim | CCW condition: Sensory - Deafness and Hearing Impairment | 0=absent, 1=present |
| ccw_hepv | CCW condition: Viral Hepatitis (General) | 0=absent, 1=present |
| ccw_hipfx | CCW condition: Hip / Pelvic Fracture | 0=absent, 1=present |
| ccw_hiv | CCW condition: Human Immunodeficiency Virus and/or Acquired Immunodeficiency Syndrome (HIV/AIDS) | 0=absent, 1=present |
| ccw_hyperten | CCW condition: Hypertension | 0=absent, 1=present |
| ccw_hypothy | CCW condition: Acquired Hypothyroidism | 0=absent, 1=present |
| ccw_hyprlip | CCW condition: Hyperlipidemia | 0=absent, 1=present |
| ccw_intdis | CCW condition: Intellectual Disabilities and Related Conditions | 0=absent, 1=present |
| ccw_ischhd | CCW condition: Ischemic Heart Disease | 0=absent, 1=present |
| ccw_leadis | CCW condition: Learning Disabilities | 0=absent, 1=present |
| ccw_leukly | CCW condition: Leukemias and Lymphomas | 0=absent, 1=present |
| ccw_liver | CCW condition: Liver Disease, Cirrhosis and Other Liver Conditions | 0=absent, 1=present |
| ccw_migr | CCW condition: Migraine and Chronic Headache | 0=absent, 1=present |
| ccw_mobimp | CCW condition: Mobility Impairments | 0=absent, 1=present |
| ccw_mulscl | CCW condition: Multiple Sclerosis and Transverse Myelitis | 0=absent, 1=present |
| ccw_musdys | CCW condition: Muscular Dystrophy | 0=absent, 1=present |
| ccw_obesty | CCW condition: Obesity | 0=absent, 1=present |
| ccw_osteo | CCW condition: Osteoporosis | 0=absent, 1=present |
| ccw_othdel | CCW condition: Other Developmental Delays | 0=absent, 1=present |
| ccw_psds | CCW condition: Personality Disorders | 0=absent, 1=present |
| ccw_ptra | CCW condition: Post-Traumatic Stress Disorder (PTSD) | 0=absent, 1=present |
| ccw_pvd | CCW condition: Peripheral Vascular Disease (PVD) | 0=absent, 1=present |
| ccw_schi | CCW condition: Schizophrenia | 0=absent, 1=present |
| ccw_schiot | CCW condition: Schizophrenia and Other Psychotic Disorders | 0=absent, 1=present |
| ccw_spibif | CCW condition: Spina Bifida and Other Congenital Anomalies of the Nervous System | 0=absent, 1=present |
| ccw_spiinj | CCW condition: Spinal Cord Injury | 0=absent, 1=present |
| ccw_stroke | CCW condition: Stroke / Transient Ischemic Attack | 0=absent, 1=present |
| ccw_toba | CCW condition: Tobacco Use | 0=absent, 1=present |
| ccw_ulcers | CCW condition: Pressure and Chronic Ulcers | 0=absent, 1=present |
| ccw_visual | CCW condition: Sensory - Blindness and Visual Impairment | 0=absent, 1=present |
| prioryred | Prior 12 months emergency department visits | Count |
| prioryrinpt | Prior 12 months inpatient hospital visits | Count |
| pdeculc | Prior 12 months decubitus ulcer | 0=absent, 1=present |
| pdem | Prior 12 months dementia primary position | 0=absent, 1=present |
| ptransfus | Prior 12 months transfusion | 0=absent, 1=present |
| pwellness | Prior 12 months annual wellness visit | 0=absent, 1=present |
| pmohs | Prior 12 months Mohs surgery | 0=absent, 1=present |
| pvisual | Prior 12 months visual exam | 0=absent, 1=present |
| pflu | Prior 12 months influenza vaccination | 0=absent, 1=present |
| pzoster | Prior 12 months herpes zoster vaccination | 0=absent, 1=present |
| ppelvic | Prior 12 months pelvic exam | 0=absent, 1=present |
| pcolon | Prior 12 months colon screening | 0=absent, 1=present |
| **Hospital Characteristics** | | |
| teachinghosp | Teaching hospital | 0=no, 1=yes |
| critaccesshosp | Critical access hospital | 0=no, 1=yes |
| nhospbedcat | Hospital beds category | 1-149, 150-299, 300-449, 450-599, 600+ |
| **Regional Characteristics** | | |
| nhhs | Department of Health and Human Services Region | Region 1 – Boston, Region 2 – New York, Region 3 – Philadelphia, Region 4 – Atlanta, Region 5 – Chicago, Region 6 – Dallas, Region 7 – Kansas City, Region 8 – Denver, Region 9 – San Francisco, Region 10 – Seattle |
| nstpdmp | State Prescription Drug Monitoring Program (PDMP) category | No PDMP, Low rigor PDMP, High rigor PDMP |

**Abbreviations:** THA/TKA, total hip or knee arthroplasty; COX, cyclooxygenase; ACE, angiotensin-converting enzyme; ARBs, angiotensin II receptor blocker; 5-HT, 5-hydroxytryptamine (serotonin); CCBs, calcium channel blockers; NDRIs, norepinephrine dopamine reuptake inhibitor; MAO, monoamine oxidase; SNRIs, serotonin norepinephrine reuptake inhibitors; SSRIs, selective serotonin reuptake inhibitor; TCAs, tricyclic antidepressants; CCW, Chronic Conditions Data Warehouse; PDMP, Prescription Drug Monitoring Program; HHS, Health and Human Services.

**Additional Table 6.** Time (in days) to treatment initiation (from hospital discharge date to first observed dispensing of an opioid of interest) across exposure groups.

|  | **Days to Treatment Initiation** | |
| --- | --- | --- |
| **Treatment** | **Mean (SD)** | **Minimum, Maximum** |
| Tramadol | 5.6 (2.5) | 0, 7 |
| Oxycodone | 4.0 (3.9) | 0, 7 |
| Hydrocodone | 4.0 (4.0) | 0, 7 |

**Abbreviations:** SD, standard deviation.

**Additional Table 7.** Censoring events other than outcomes across treatment groups.

| **Censoring Reason** | **Tramadol**  **(n=2,697)** | **Oxycodone**  **(n=11,407)** | **Hydrocodone**  **(n=14,665)** |
| --- | --- | --- | --- |
| **All-cause SAEs, without drug adherence censoring** | | | |
| Death, n (%) | 12 (0.44) | 49 (0.43) | 56 (0.38) |
| Administrative end of follow-up, n (%) | 2,222 (82.39) | 9,557 (83.78) | 12,375 (84.38) |
| HMO enrollment, n (%) | 49 (1.82) | 217 (1.90) | 274 (1.87) |
| Part D disenrollment, n (%) | 11 (0.41) | 60 (0.53) | 94 (0.64) |
| **All-cause SAEs, with drug adherence censoring** | | | |
| Death, n (%) | *** | *** | *** |
| Administrative end of follow-up, n (%) | 178 (6.60) | 626 (5.49) | 762 (5.2) |
| HMO enrollment, n (%) | *** | 23 (0.20) | 39 (0.27) |
| Medication switching or discontinuation, n (%) | 2,237 (82.94) | 9,697 (85.01) | 12,568 (85.70) |
| Part D disenrollment, n (%) | *** | *** | *** |
| **Composite SAEs, without drug adherence censoring** | | | |
| Death, n (%) | 14 (0.52) | 71 (0.62) | 80 (0.55) |
| Administrative end of follow-up, n (%) | 2,529 (93.77) | 10,711 (93.90) | 13,753 (93.78) |
| HMO enrollment, n (%) | 57 (2.11) | 238 (2.09) | 310 (2.11) |
| Part D disenrollment, n (%) | 14 (0.52) | 72 (0.63) | 105 (0.72) |
| **All-cause SAEs, with drug adherence censoring** | | | |
| Death, n (%) | *** | *** | *** |
| Administrative end of follow-up, n (%) | 163 (6.04) | 627 (5.50) | 721 (4.92) |
| HMO enrollment, n (%) | *** | 24 (0.21) | 38 (0.26) |
| Medication switching or discontinuation, n (%) | 2,474 (91.73) | 10,564 (92.61) | 13,631 (92.95) |
| Part D disenrollment, n (%) | *** | *** | *** |

**Abbreviations:** HMO, Health Maintenance Organization; SAE, serious adverse events.

**Note:** *** due to data use agreements with the Centers for Medicare and Medicaid that prohibit the reporting of small cells.

**Additional Table 8.** Absolute standardized differences prior to and after inverse probability of treatment weighting.

| **Variable^a^** | **Hydrocodone vs. Oxycodone** | | **Hydrocodone vs. Tramadol** | | **Oxycodone vs. Tramadol** | |
| --- | --- | --- | --- | --- | --- | --- |
|  | **Unweighted** | **Weighted** | **Unweighted** | **Weighted** | **Unweighted** | **Weighted** |
| Summary |  |  |  |  |  |  |
| Minimum | 0.000 | 0.000 | 0.000 | 0.000 | 0.000 | 0.000 |
| Maximum | 0.560 | 0.085 | 0.300 | 0.063 | 0.328 | 0.058 |
| Mean | 0.045 | 0.009 | 0.063 | 0.019 | 0.056 | 0.019 |
| Standard Deviation | 0.073 | 0.009 | 0.062 | 0.014 | 0.065 | 0.014 |
|  |  |  |  |  |  |  |
| prioryred | 0.022 | 0.01 | 0.079 | 0.009 | 0.057 | 0.019 |
| prioryrinpt | 0.003 | 0.004 | 0.003 | 0.043 | 0.001 | 0.047 |
| numpriormed | 0.005 | 0.002 | 0.047 | 0.036 | 0.051 | 0.038 |
| ccw_acp | 0.01 | 0.006 | 0.013 | 0.018 | 0.003 | 0.023 |
| ccw_alz | 0.025 | 0.014 | 0.004 | 0.012 | 0.028 | 0.003 |
| ccw_alzdem | 0.025 | 0.007 | 0.057 | 0.014 | 0.082 | 0.021 |
| ccw_ami | 0.004 | 0.012 | 0.033 | 0.001 | 0.03 | 0.013 |
| ccw_anem | 0.008 | 0.009 | 0.14 | 0.018 | 0.132 | 0.028 |
| ccw_anxi | 0.005 | 0.002 | 0.062 | 0.018 | 0.057 | 0.016 |
| ccw_arthr | 0.005 | 0.001 | 0.013 | 0.011 | 0.017 | 0.01 |
| ccw_asth | 0.013 | 0.002 | 0.063 | 0.01 | 0.05 | 0.012 |
| ccw_atrfib | 0.017 | 0.006 | 0.065 | 0.001 | 0.048 | 0.005 |
| ccw_bipl | 0.014 | 0.011 | 0.014 | 0.006 | 0.001 | 0.017 |
| ccw_bph | 0.019 | 0.011 | 0.084 | 0.03 | 0.102 | 0.041 |
| ccw_brainj | 0.008 | 0.007 | 0.016 | 0.015 | 0.008 | 0.022 |
| ccw_cabrst | 0.009 | 0.004 | 0.1 | 0.003 | 0.11 | 0 |
| ccw_cacolo | 0.02 | 0.008 | 0.014 | 0.022 | 0.006 | 0.014 |
| ccw_caendo | 0.003 | 0.002 | 0.01 | 0.005 | 0.013 | 0.003 |
| ccw_calung | 0.013 | 0.013 | 0.021 | 0.006 | 0.034 | 0.007 |
| ccw_capros | 0.015 | 0.008 | 0.024 | 0.006 | 0.039 | 0.014 |
| ccw_catar | 0.062 | 0.014 | 0.175 | 0.029 | 0.237 | 0.043 |
| ccw_cerpal | 0.024 | 0.021 | 0.019 | 0.044 | 0.004 | 0.023 |
| ccw_chf | 0.025 | 0.015 | 0.086 | 0.004 | 0.111 | 0.012 |
| ccw_chrkid | 0.035 | 0.007 | 0.077 | 0.002 | 0.042 | 0.005 |
| ccw_copd | 0.021 | 0.009 | 0.029 | 0.03 | 0.05 | 0.021 |
| ccw_cysfib | 0.01 | 0 | 0.028 | 0.004 | 0.018 | 0.004 |
| ccw_depr | 0.013 | 0.003 | 0.044 | 0 | 0.031 | 0.003 |
| ccw_depsn | 0.007 | 0.005 | 0.036 | 0.014 | 0.029 | 0.009 |
| ccw_diab | 0.013 | 0.001 | 0 | 0.029 | 0.013 | 0.031 |
| ccw_epilep | 0.009 | 0.006 | 0.008 | 0.027 | 0.001 | 0.021 |
| ccw_fibro | 0.014 | 0.001 | 0.094 | 0.002 | 0.108 | 0.002 |
| ccw_glauc | 0.026 | 0.002 | 0.113 | 0.007 | 0.087 | 0.008 |
| ccw_hearim | 0.019 | 0.001 | 0.129 | 0.012 | 0.11 | 0.014 |
| ccw_hepv | 0.046 | 0.013 | 0.04 | 0.01 | 0.006 | 0.003 |
| ccw_hipfx | 0.029 | 0.003 | 0.046 | 0.006 | 0.076 | 0.003 |
| ccw_hiv | 0 | 0.002 | 0.026 | 0.022 | 0.026 | 0.025 |
| ccw_hyperten | 0.004 | 0.008 | 0.005 | 0.031 | 0.001 | 0.039 |
| ccw_hypothy | 0.02 | 0.004 | 0.129 | 0.004 | 0.149 | 0.008 |
| ccw_hyprlip | 0.035 | 0.005 | 0.04 | 0.011 | 0.005 | 0.015 |
| ccw_intdis | 0 | 0 | 0.026 | 0.023 | 0.026 | 0.022 |
| ccw_ischhd | 0.021 | 0.01 | 0.04 | 0.012 | 0.061 | 0.002 |
| ccw_leadis | 0.017 | 0.031 | 0.036 | 0.059 | 0.019 | 0.028 |
| ccw_leukly | 0.015 | 0.005 | 0.022 | 0.024 | 0.007 | 0.019 |
| ccw_liver | 0.008 | 0.006 | 0.038 | 0.005 | 0.03 | 0 |
| ccw_migr | 0.001 | 0.004 | 0.039 | 0.011 | 0.039 | 0.015 |
| ccw_mobimp | 0.001 | 0.004 | 0.02 | 0.008 | 0.021 | 0.012 |
| ccw_mulscl | 0.012 | 0.004 | 0.001 | 0.018 | 0.013 | 0.022 |
| ccw_musdys | 0.001 | 0.006 | 0.021 | 0.016 | 0.022 | 0.021 |
| ccw_obesty | 0.057 | 0.015 | 0.026 | 0.008 | 0.031 | 0.024 |
| ccw_osteo | 0.041 | 0.005 | 0.174 | 0.011 | 0.215 | 0.016 |
| ccw_othdel | 0.002 | 0.002 | 0.008 | 0.006 | 0.011 | 0.008 |
| ccw_psds | 0.016 | 0.018 | 0.015 | 0.009 | 0.03 | 0.009 |
| ccw_ptra | 0.015 | 0.005 | 0.014 | 0.009 | 0.029 | 0.004 |
| ccw_pvd | 0.004 | 0.004 | 0.068 | 0.024 | 0.064 | 0.029 |
| ccw_schi | 0.002 | 0.003 | 0.027 | 0.027 | 0.025 | 0.024 |
| ccw_schiot | 0.004 | 0.005 | 0.016 | 0.017 | 0.02 | 0.022 |
| ccw_spibif | 0.022 | 0.012 | 0.015 | 0.001 | 0.007 | 0.013 |
| ccw_spiinj | 0.009 | 0.012 | 0.011 | 0.004 | 0.02 | 0.008 |
| ccw_stroke | 0.02 | 0.009 | 0.065 | 0.017 | 0.086 | 0.026 |
| ccw_toba | 0.009 | 0 | 0.048 | 0.058 | 0.057 | 0.058 |
| ccw_ulcers | 0.02 | 0.01 | 0.027 | 0.026 | 0.007 | 0.036 |
| ccw_visual | 0.01 | 0.002 | 0.047 | 0.011 | 0.057 | 0.009 |
| pre180benzo_Max | 0.039 | 0.005 | 0.071 | 0.001 | 0.032 | 0.006 |
| pre180cox2_Max | 0.016 | 0.02 | 0.008 | 0.006 | 0.024 | 0.026 |
| pre180semicox_Max | 0.019 | 0.004 | 0.029 | 0.005 | 0.048 | 0.001 |
| pre180noncox_Max | 0.003 | 0.001 | 0.071 | 0.042 | 0.074 | 0.041 |
| pre180apap_Max | 0.036 | 0.007 | 0.066 | 0.025 | 0.031 | 0.032 |
| pre180irrevcox_Max | 0.006 | 0.013 | 0.016 | 0.016 | 0.011 | 0.003 |
| pre180ppi_Max | 0.01 | 0.001 | 0.057 | 0.003 | 0.047 | 0.004 |
| pre180gabas_Max | 0.002 | 0.003 | 0.012 | 0.017 | 0.014 | 0.02 |
| pre180anticonv_Max | 0.019 | 0.002 | 0.013 | 0.035 | 0.006 | 0.038 |
| pre180musrelax_Max | 0.007 | 0.004 | 0.014 | 0.003 | 0.022 | 0.007 |
| pre180abx_Max | 0.017 | 0.002 | 0.045 | 0.021 | 0.062 | 0.023 |
| pre180ace_Max | 0.017 | 0.002 | 0.039 | 0.022 | 0.022 | 0.021 |
| pre180antiplate_Max | 0.009 | 0.003 | 0.006 | 0.003 | 0.015 | 0.006 |
| pre180antixa_Max | 0.007 | 0.011 | 0.003 | 0.022 | 0.011 | 0.011 |
| pre180arb_Max | 0.027 | 0.004 | 0.008 | 0.021 | 0.018 | 0.025 |
| pre180beta_Max | 0.021 | 0.007 | 0.064 | 0.002 | 0.085 | 0.009 |
| pre180cortico_Max | 0.008 | 0.005 | 0.023 | 0.018 | 0.015 | 0.013 |
| pre180dhccb_Max | 0.012 | 0.01 | 0.025 | 0.031 | 0.013 | 0.02 |
| pre180hypnotics_Max | 0.016 | 0.001 | 0.033 | 0.036 | 0.049 | 0.037 |
| pre180insulin_Max | 0.02 | 0.012 | 0.037 | 0.028 | 0.017 | 0.016 |
| pre180ksparing_Max | 0.006 | 0.007 | 0.016 | 0.011 | 0.01 | 0.018 |
| pre180loop_Max | 0.031 | 0.013 | 0.024 | 0.029 | 0.055 | 0.015 |
| pre180maoi_Max | 0.002 | 0.001 | 0.005 | 0.006 | 0.003 | 0.005 |
| pre180mirtaz_Max | 0.001 | 0.004 | 0.036 | 0.015 | 0.035 | 0.019 |
| pre180miso_Max | 0.002 | 0.006 | 0.009 | 0.011 | 0.011 | 0.017 |
| pre180mixed5ht_Max | 0.011 | 0.004 | 0.03 | 0.034 | 0.041 | 0.037 |
| pre180ndri_Max | 0.025 | 0.004 | 0.018 | 0.022 | 0.043 | 0.026 |
| pre180nitrate_Max | 0.022 | 0.007 | 0.015 | 0.042 | 0.006 | 0.035 |
| pre180nondhccb_Max | 0.011 | 0.002 | 0.004 | 0.023 | 0.015 | 0.025 |
| pre180noninsdiab_Max | 0.021 | 0.012 | 0.069 | 0.063 | 0.048 | 0.051 |
| pre180osteopor_Max | 0.038 | 0.007 | 0.028 | 0.013 | 0.066 | 0.006 |
| pre180snri_Max | 0.001 | 0.01 | 0.005 | 0.036 | 0.005 | 0.026 |
| pre180ssri_Max | 0.004 | 0.001 | 0.059 | 0.056 | 0.063 | 0.055 |
| pre180statin_Max | 0.019 | 0.003 | 0.035 | 0.027 | 0.054 | 0.03 |
| pre180tca_Max | 0.007 | 0.009 | 0.007 | 0.028 | 0 | 0.037 |
| pre180thiazide_Max | 0.024 | 0.009 | 0.071 | 0.035 | 0.047 | 0.026 |
| pre180warfarin_Max | 0.038 | 0.007 | 0.028 | 0.004 | 0.066 | 0.011 |
| pdeculc | 0.003 | 0.003 | 0.025 | 0.008 | 0.022 | 0.004 |
| pdem | 0.006 | 0.006 | 0.033 | 0.002 | 0.028 | 0.003 |
| pwellness | 0.105 | 0.018 | 0.132 | 0.034 | 0.027 | 0.015 |
| paccident | 0.02 | 0.013 | 0.078 | 0.002 | 0.058 | 0.011 |
| pmohs | 0.034 | 0.013 | 0.045 | 0.005 | 0.01 | 0.008 |
| pvisual | 0.027 | 0.009 | 0.016 | 0.024 | 0.011 | 0.014 |
| pflu | 0.005 | 0.001 | 0.015 | 0.002 | 0.02 | 0.001 |
| pzoster | 0.033 | 0.008 | 0.002 | 0.009 | 0.035 | 0.017 |
| ppelvic | 0.019 | 0.012 | 0.076 | 0.023 | 0.057 | 0.011 |
| pcolon | 0.027 | 0.01 | 0.003 | 0.013 | 0.03 | 0.003 |
| thatkayear:2010 | 0.16 | 0.017 | 0.277 | 0.057 | 0.117 | 0.04 |
| thatkayear:2011 | 0.128 | 0.027 | 0.125 | 0.012 | 0.003 | 0.015 |
| thatkayear:2012 | 0.026 | 0.007 | 0.011 | 0.007 | 0.015 | 0 |
| thatkayear:2013 | 0.01 | 0.002 | 0.065 | 0.012 | 0.075 | 0.01 |
| thatkayear:2014 | 0.088 | 0.015 | 0.115 | 0.023 | 0.028 | 0.008 |
| thatkayear:2015 | 0.194 | 0.027 | 0.174 | 0.03 | 0.02 | 0.003 |
| pxqrtr:1 | 0.013 | 0.01 | 0.084 | 0.023 | 0.071 | 0.013 |
| pxqrtr:2 | 0.001 | 0.006 | 0.05 | 0.018 | 0.049 | 0.012 |
| pxqrtr:3 | 0.007 | 0.007 | 0.065 | 0.017 | 0.058 | 0.023 |
| pxqrtr:4 | 0.006 | 0.012 | 0.032 | 0.012 | 0.038 | 0.024 |
| hksex:1 | 0.079 | 0.013 | 0.249 | 0.023 | 0.328 | 0.036 |
| hksex:2 | 0.079 | 0.013 | 0.249 | 0.023 | 0.328 | 0.036 |
| nagecat:Age: 65-74 | 0.11 | 0.022 | 0.207 | 0.003 | 0.317 | 0.025 |
| nagecat:Age: 75-84 | 0.095 | 0.017 | 0.14 | 0.001 | 0.235 | 0.017 |
| nagecat:Age: 85+ | 0.044 | 0.012 | 0.184 | 0.011 | 0.228 | 0.022 |
| nrace:Race/ethnicity: Black-NH | 0.064 | 0.009 | 0.022 | 0.034 | 0.086 | 0.043 |
| nrace:Race/ethnicity: Hispanic | 0.065 | 0.002 | 0.06 | 0.034 | 0.005 | 0.032 |
| nrace:Race/ethnicity: Other | 0.039 | 0.011 | 0.008 | 0.007 | 0.047 | 0.004 |
| nrace:Race/ethnicity: White-NH | 0.028 | 0.011 | 0.051 | 0.036 | 0.08 | 0.047 |
| nhipknee:TotalHip | 0.009 | 0.001 | 0.155 | 0.02 | 0.146 | 0.021 |
| nhipknee:TotalKnee | 0.009 | 0.001 | 0.155 | 0.02 | 0.146 | 0.021 |
| dualany12pre:0 | 0.022 | 0.009 | 0.068 | 0.043 | 0.046 | 0.035 |
| dualany12pre:1 | 0.022 | 0.009 | 0.068 | 0.043 | 0.046 | 0.035 |
| nfrailtycat:Frailty:Mildly Frail | 0 | 0 | 0.046 | 0.002 | 0.046 | 0.002 |
| nfrailtycat:Frailty:Prefail | 0.035 | 0.008 | 0.075 | 0.035 | 0.109 | 0.026 |
| nfrailtycat:Frailty:Robust | 0.035 | 0.008 | 0.079 | 0.035 | 0.114 | 0.027 |
| ncomorbidcat:Comorbid:<0 | 0.006 | 0.011 | 0.084 | 0.022 | 0.079 | 0.033 |
| ncomorbidcat:Comorbid:0 | 0.018 | 0.007 | 0.058 | 0.006 | 0.039 | 0.002 |
| ncomorbidcat:Comorbid:1 | 0.004 | 0.004 | 0.04 | 0.012 | 0.035 | 0.015 |
| ncomorbidcat:Comorbid:2 | 0.002 | 0.005 | 0.031 | 0.008 | 0.029 | 0.013 |
| ncomorbidcat:Comorbid:3+ | 0.024 | 0.005 | 0.099 | 0.012 | 0.075 | 0.007 |
| Alcohol_abuse:0 | 0.033 | 0.016 | 0.009 | 0.008 | 0.025 | 0.008 |
| Alcohol_abuse:1 | 0.033 | 0.016 | 0.009 | 0.008 | 0.025 | 0.008 |
| cancerflag:0 | 0.029 | 0.012 | 0.046 | 0.01 | 0.017 | 0.002 |
| cancerflag:1 | 0.029 | 0.012 | 0.046 | 0.01 | 0.017 | 0.002 |
| nhhs:HHS 1-Boston | 0.238 | 0.025 | 0.13 | 0.008 | 0.108 | 0.017 |
| nhhs:HHS 10-Seattle | 0.097 | 0.008 | 0.033 | 0.009 | 0.131 | 0.017 |
| nhhs:HHS 2-New York | 0.242 | 0.026 | 0.248 | 0.021 | 0.006 | 0.004 |
| nhhs:HHS 3-Philadelphia | 0.179 | 0.015 | 0.15 | 0.007 | 0.029 | 0.008 |
| nhhs:HHS 4-Atlanta | 0.191 | 0.011 | 0.124 | 0.033 | 0.315 | 0.044 |
| nhhs:HHS 5-Chicago | 0.234 | 0.009 | 0.092 | 0 | 0.142 | 0.009 |
| nhhs:HHS 6-Dallas | 0.56 | 0.085 | 0.3 | 0.034 | 0.26 | 0.051 |
| nhhs:HHS 7-Kansas City | 0.236 | 0.008 | 0.012 | 0.025 | 0.225 | 0.032 |
| nhhs:HHS 8-Denver | 0.07 | 0.003 | 0.12 | 0.023 | 0.19 | 0.026 |
| nhhs:HHS 9-San Francisco | 0.041 | 0.001 | 0.094 | 0.003 | 0.053 | 0.004 |
| nstpdmp:State PDMP: High rigor | 0.256 | 0.022 | 0.168 | 0.016 | 0.088 | 0.038 |
| nstpdmp:State PDMP: Low rigor | 0.01 | 0.019 | 0.003 | 0.008 | 0.013 | 0.027 |
| nstpdmp:State PDMP: None | 0.206 | 0.001 | 0.145 | 0.005 | 0.061 | 0.004 |
| teachinghosp:0 | 0.171 | 0.015 | 0.157 | 0.044 | 0.015 | 0.029 |
| teachinghosp:1 | 0.171 | 0.015 | 0.157 | 0.044 | 0.015 | 0.029 |
| critaccesshosp:0 | 0.011 | 0.005 | 0.084 | 0.046 | 0.073 | 0.051 |
| critaccesshosp:1 | 0.011 | 0.005 | 0.084 | 0.046 | 0.073 | 0.051 |
| nhospbedcat:Beds: 0-149 | 0.158 | 0.005 | 0.135 | 0.038 | 0.023 | 0.043 |
| nhospbedcat:Beds: 150-299 | 0.012 | 0.007 | 0.016 | 0.01 | 0.005 | 0.003 |
| nhospbedcat:Beds: 300-449 | 0.036 | 0.006 | 0.008 | 0.016 | 0.044 | 0.022 |
| nhospbedcat:Beds: 450-599 | 0.07 | 0.012 | 0.09 | 0.017 | 0.02 | 0.005 |
| nhospbedcat:Beds: 600+ | 0.181 | 0.001 | 0.097 | 0.025 | 0.085 | 0.026 |
| tramadoldea:0 | 0.225 | 0.027 | 0.209 | 0.036 | 0.016 | 0.009 |
| tramadoldea:1 | 0.225 | 0.027 | 0.209 | 0.036 | 0.016 | 0.009 |
| hydrodea:0 | 0.225 | 0.029 | 0.194 | 0.039 | 0.032 | 0.01 |
| hydrodea:1 | 0.225 | 0.029 | 0.194 | 0.039 | 0.032 | 0.01 |
| loscat:1 | 0.054 | 0 | 0.031 | 0.008 | 0.023 | 0.007 |
| loscat:2 | 0.061 | 0.013 | 0.031 | 0.022 | 0.092 | 0.035 |
| loscat:3 | 0.087 | 0.012 | 0.012 | 0.026 | 0.1 | 0.038 |
| ^a^Descriptions of covariate names are provided in Additional Table 5. | | | | | | |

**Additional Table 9.** Stabilized inverse probability of treatment and censoring weight distributions.

| **Pairwise Comparisons** | **Mean (SD)** | **Median (Q1, Q3)** | **Minimum, Maximum** |
| --- | --- | --- | --- |
| **GBM estimation** | | | |
| IPTW | 0.95 (0.47) | 0.81 (0.68, 1.07) | 0.12, 7.44 |
| Per-protocol IPTWxIPCW product (99^th^ percentile truncated) | Inpt-ED: 0.93 (0.49)  Any SAE: 0.95 (0.62) | Inpt-ED: 0.79 (0.65, 1.07)  Any SAE: 0.77 (0.60, 1.08) | Inpt-ED: 0.09, 3.27  Any SAE: 0.08, 4.08 |
| **Multinomial logistic estimation** | | | |
| IPTW | 1.00 (0.55) | 0.84 (0.69, 1.11) | 0.16, 10.83 |
| IPCW | Inpt-ED: 1.05 (3.28)  Any SAE: 1.05 (2.34) | Inpt-ED: 0.97 (0.83, 1.02)  Any SAE: 0.97 (0.84, 1.02) | Inpt-ED: 0.00, 1988.93  Any SAE: 0.00, 1459.18 |
| Per-protocol IPTWxIPCW product (99^th^ percentile truncated) | Inpt-ED: 1.00 (0.67)  Any SAE: 1.00 (0.68) | Inpt-ED: 0.80 (0.62, 1.12)  Any SAE: 0.80 (0.62, 1.12) | Inpt-ED: 0.13, 4.46  Any SAE: 0.11, 4.51 |

**Abbreviations:** SD, standard deviation; Q1, first quartile; Q3, third quartile; GBM, Generalized Boosted Regression Model; IPTW, inverse probability of treatment weights; IPCW, inverse probability of censoring weights; Inpt, inpatient; ED, emergency department; SAE, serious adverse events.

**Additional Table 10.** Observed 90-day follow-up times and outcomes stratified by postoperative treatment.

| **Outcomes** | **Tramadol**  **(n=2,697)** | **Oxycodone**  **(n=11,407)** | **Hydrocodone**  **(n=14,665)** |
| --- | --- | --- | --- |
| **Follow-up time without drug adherence censoring (intention-to-treat), mean (SD), days** | | | |
| All-cause SAEs | 76.0 (28.4) | 78.0 (26.4) | 79.5 (24.8) |
| Composite SAEs | 84.7 (18.0) | 85.4 (16.6) | 86.5 (14.7) |
| **Follow-up time with drug adherence censoring (per-protocol), mean (SD), days** | | | |
| All-cause SAEs | 26.0 (22.2) | 25.8 (23.5) | 29.2 (24.4) |
| Composite SAEs | 28.5 (23.26) | 28.0 (24.7) | 31.3 (25.4) |
| **Primary outcomes without drug adherence censoring (intention-to-treat), n (%)** | | | |
| All-cause SAEs | 403 (14.9) | 1,524 (13.4) | 1,866 (12.7) |
| Composite SAEs | 83 (3.1) | 315 (2.8) | 417 (2.8) |
| **Primary outcomes with drug adherence censoring (per-protocol), n (%)** | | | |
| All-cause SAEs | 278 (10.3) | 1,048 (9.2) | 1,283 (8.7) |
| Composite SAEs | 54 (2.0) | 174 (1.5) | 256 (1.7) |

**Note:** Outcome definitions for SAEs are listed in Additional Table 4.

**Abbreviations:** SD, standard deviation; ED, emergency department; SAEs, serious adverse events.

**Additional Table 11.** Numbers needed to treat and harm for 90-day outcomes comparing postoperative tramadol use to oxycodone and hydrocodone.

| **Comparisons** | **90-day All-cause SAE** | **90-day Composite SAE** |
| --- | --- | --- |
|  | **NNH/NNT (95% CLs)** | **NNH/NNT (95% CLs)** |
| **Oxycodone as comparator** | | |
| Unadjusted | NNH: 51 (322, 28) | NNH: 285 (NNT 244 to ∞ to NNH 92) |
| Intention-to-treat | NNH: 128 (NNT 100 to ∞ to NNH 38) | NNH: 715 (NNT 152 to ∞ to NNH 94) |
| Per-protocol | NNH: 205 (NNT 18 to ∞ to NNH 17) | NNH: 33 (NNT 209 to ∞ to NNH 12) |
| **Hydrocodone as comparator** | | |
| Unadjusted | NNH: 35 (75, 22) | NNH: 333 (NNT 250 to ∞ to NNH 104) |
| Intention-to-treat | NNH: 90 (NNT 156 to ∞ to NNH 36) | NNH: 2000 (NNT 141 to ∞ to NNH 119) |
| Per-protocol | NNH: 25 (NNT 24 to ∞ to NNH 8) | NNH: 28 (NNT 114 to ∞ to NNH 11) |

**Notes:** Estimates derived from standardized survival curves with 95% CL estimated using the percentile-method from 500 bootstrap replications. Estimated using 1/(treated survival-control survival). Non-significant 95% Confidence Limits reported using the method recommended by Altman.^20^ Outcome definitions for SAEs are listed in Additional Table 4.

**Abbreviations**: ED, emergency department; SAE, serious adverse events; NNH, number needed to harm; NNT, number needed to treat; CLs, confidence limits.

**Additional Table 12.** E-values for formal quantitative bias analyses.

| **Comparisons** | **90-day All-cause SAE** | | **90-day Composite SAE** | |
| --- | --- | --- | --- | --- |
|  | **HR (95% CLs)** | **E-Value,**  **Point Estimate (Lower Limit)** | **HR (95% CLs)** | **E-Value,**  **Point Estimate (Lower Limit)** |
| **Oxycodone as comparator** | | | | |
| Unadjusted | 1.34* (1.17, 1.53) | 2.01 (1.62) | 1.15 (0.84, 1.57) | 1.57 (1**) |
| Intention-to-treat | 1.19* (1.02, 1.41) | 1.67 (1.16) | 1.12 (0.78, 1.64) | 1.49 (1**) |
| Per-Protocol | 1.05 (0.86, 1.29) | 1.28 (1**) | 1.49 (0.95, 2.35) | 2.34 (1**) |
| **Hydrocodone as comparator** | | | | |
| Unadjusted | 1.69* (1.38, 2.06) | 2.77 (2.10) | 1.15 (0.72, 1.83) | 1.57 (1**) |
| Intention-to-treat | 1.40* (1.10, 1.76) | 2.15 (1.43) | 1.19 (0.69, 2.04) | 1.67 (1**) |
| Per-Protocol | 1.34* (1.03, 1.75) | 2.01 (1.21) | 1.63 (0.90, 2.95) | 2.64 (1**) |

**Note:** Outcome definitions for SAEs are listed in Additional Table 4.

**Abbreviations:** ED, emergency department; SAE, Serious Adverse Events; HR, hazard ratio; CLs, confidence limits.

*p<0.05

**Value is 1 because HR 95% CI estimate includes 1.

**SUPPLEMENTARY REFERENCES**

1. McCaffrey DF, Griffin BA, Almirall D, Slaughter ME, Ramchand R, Burgette LF. A tutorial on propensity score estimation for multiple treatments using generalized boosted models. *Stat Med*. Aug 30 2013;32(19):3388-414. doi:10.1002/sim.5753

2. Austin PC. An Introduction to Propensity Score Methods for Reducing the Effects of Confounding in Observational Studies. *Multivariate behavioral research*. May 2011;46(3):399-424. doi:10.1080/00273171.2011.568786

3. Schneeweiss S, Robicsek A, Scranton R, Zuckerman D, Solomon DH. Veteran's affairs hospital discharge databases coded serious bacterial infections accurately. *J Clin Epidemiol*. Apr 2007;60(4):397-409. doi:10.1016/j.jclinepi.2006.07.011

4. Olsen MA, Ball KE, Nickel KB, Wallace AE, Fraser VJ. Validation of ICD-9-CM Diagnosis Codes for Surgical Site Infection and Noninfectious Wound Complications After Mastectomy. *Infection control and hospital epidemiology*. Mar 2017;38(3):334-339. doi:10.1017/ice.2016.271

5. Cutrona SL, Toh S, Iyer A, et al. Validation of acute myocardial infarction in the Food and Drug Administration's Mini-Sentinel program. *Pharmacoepidemiology and drug safety*. Jan 2013;22(1):40-54. doi:10.1002/pds.3310

6. Kumamaru H, Judd SE, Curtis JR, et al. Validity of claims-based stroke algorithms in contemporary Medicare data: reasons for geographic and racial differences in stroke (REGARDS) study linked with medicare claims. *Circulation Cardiovascular quality and outcomes*. Jul 2014;7(4):611-9. doi:10.1161/circoutcomes.113.000743

7. Saczynski JS, Andrade SE, Harrold LR, et al. A systematic review of validated methods for identifying heart failure using administrative data. *Pharmacoepidemiol Drug Saf*. Jan 2012;21 Suppl 1:129-40. doi:10.1002/pds.2313

8. Singh S, Fouayzi H, Anzuoni K, et al. Diagnostic Algorithms for Cardiovascular Death in Administrative Claims Databases: A Systematic Review. *Drug safety*. Apr 2019;42(4):515-527. doi:10.1007/s40264-018-0754-z

9. Curtis JR, Mudano AS, Solomon DH, Xi J, Melton ME, Saag KG. Identification and validation of vertebral compression fractures using administrative claims data. *Medical care*. Jan 2009;47(1):69-72. doi:10.1097/MLR.0b013e3181808c05

10. Ray WA, Griffin MR, Fought RL, Adams ML. Identification of fractures from computerized Medicare files. *J Clin Epidemiol*. Jul 1992;45(7):703-14. doi:10.1016/0895-4356(92)90047-q

11. Ray WA, Chung CP, Stein CM, et al. Risk of peptic ulcer hospitalizations in users of NSAIDs with gastroprotective cotherapy versus coxibs. *Gastroenterology*. Sep 2007;133(3):790-8. doi:10.1053/j.gastro.2007.06.058

12. Abraham NS, Cohen DC, Rivers B, Richardson P. Validation of administrative data used for the diagnosis of upper gastrointestinal events following nonsteroidal anti-inflammatory drug prescription. *Aliment Pharmacol Ther*. Jul 15 2006;24(2):299-306. doi:10.1111/j.1365-2036.2006.02985.x

13. Winner M, Mooney SJ, Hershman DL, et al. Incidence and predictors of bowel obstruction in elderly patients with stage IV colon cancer: a population-based cohort study. *JAMA Surg*. Aug 2013;148(8):715-22. doi:10.1001/jamasurg.2013.1

14. Lo Re V, 3rd, Haynes K, Goldberg D, et al. Validity of diagnostic codes to identify cases of severe acute liver injury in the US Food and Drug Administration's Mini-Sentinel Distributed Database. *Pharmacoepidemiology and drug safety*. Aug 2013;22(8):861-72. doi:10.1002/pds.3470

15. Waikar SS, Wald R, Chertow GM, et al. Validity of International Classification of Diseases, Ninth Revision, Clinical Modification Codes for Acute Renal Failure. *J Am Soc Nephrol*. Jun 2006;17(6):1688-94. doi:10.1681/ASN.2006010073

16. Heslin KC, Owens PL, Karaca Z, Barrett ML, Moore BJ, Elixhauser A. Trends in Opioid-related Inpatient Stays Shifted After the US Transitioned to ICD-10-CM Diagnosis Coding in 2015. *Medical care*. 2017;55(11):918-923. doi:10.1097/MLR.0000000000000805

17. Green CA, Perrin NA, Janoff SL, Campbell CI, Chilcoat HD, Coplan PM. Assessing the accuracy of opioid overdose and poisoning codes in diagnostic information from electronic health records, claims data, and death records. *Pharmacoepidemiology and drug safety*. May 2017;26(5):509-517. doi:10.1002/pds.4157

18. Kim DH, Lee J, Kim CA, et al. Evaluation of algorithms to identify delirium in administrative claims and drug utilization database. *Pharmacoepidemiol Drug Saf*. Aug 2017;26(8):945-953. doi:10.1002/pds.4226

19. Jones N, Schneider G, Kachroo S, Rotella P, Avetisyan R, Reynolds MW. A systematic review of validated methods for identifying acute respiratory failure using administrative and claims data. *Pharmacoepidemiology and drug safety*. Jan 2012;21 Suppl 1:261-4. doi:10.1002/pds.2326

20. Altman DG. Confidence intervals for the number needed to treat. *BMJ (Clinical research ed)*. Nov 7 1998;317(7168):1309-12. doi:10.1136/bmj.317.7168.1309
